# Activation of Bone Marrow Adaptive Immunity in Type 2 Diabetes: Rescue by Co-stimulation Modulator Abatacept

**DOI:** 10.3389/fimmu.2021.609406

**Published:** 2021-03-04

**Authors:** Marianna Santopaolo, Niall Sullivan, Anita Coral Thomas, Valeria Vincenza Alvino, Lindsay B. Nicholson, Yue Gu, Gaia Spinetti, Marinos Kallikourdis, Ashley Blom, Paolo Madeddu

**Affiliations:** ^1^Bristol Medical School, Translational Health Sciences, University of Bristol, Bristol, United Kingdom; ^2^University Hospitals Bristol NHS Trust, Bristol, United Kingdom; ^3^Bristol Medical School, School of Cellular and Molecular Medicine, University of Bristol, Bristol, United Kingdom; ^4^Laboratory of Cardiovascular Research, Istituto di Ricovero e Cura a Carattere Scientifico MultiMedica, Milan, Italy; ^5^Department of Biomedical Sciences, Humanitas University, Milan, Italy; ^6^Adaptive Immunity Laboratory, IRCCS Humanitas Research Hospital, Milan, Italy

**Keywords:** diabetes, immunity, bone marrow, cardiovaacular, adipo/cytokines

## Abstract

**Background:** Chronic low-grade inflammation and alterations in innate and adaptive immunity were reported in Type 2 diabetes (T2D). Here, we investigated the abundance and activation of T cells in the bone marrow (BM) of patients with T2D. We then verified the human data in a murine model and tested if the activation of T cells can be rescued by treating mice with abatacept, an immunomodulatory drug employed for the treatment of rheumatoid arthritis. Clinical evidence indicated abatacept can slow the decline in beta-cell function.

**Methods:** A cohort of 24 patients (12 with T2D) undergoing hip replacement surgery was enrolled in the study. Flow cytometry and cytokine analyses were performed on BM leftovers from surgery. We next compared the immune profile of db/db and control wt/db mice. In an additional study, db/db mice were randomized to receive abatacept or vehicle for 4 weeks, with endpoints being immune cell profile, indices of insulin sensitivity, and heart performance.

**Results:** Patients with T2D showed increased frequencies of BM CD4^+^ (2.8-fold, *p* = 0.001) and CD8^+^ T cells (1.8-fold, *p* = 0.01), with the upregulation of the activation marker CD69 and the homing receptor CCR7 in CD4^+^ (1.64-fold, *p* = 0.003 and 2.27-fold, *p* = 0.01, respectively) and CD8^+^ fractions (1.79-fold, *p* = 0.05 and 1.69-fold, *p* = 0.02, respectively). These differences were confirmed in a multivariable regression model. CCL19 (CCR7 receptor ligand) and CXCL10/11 (CXCR3 receptor ligands), implicated in T-cell migration and activation, were the most differentially modulated chemokines. Studies in mice confirmed the activation of adaptive immunity in T2D. Abatacept reduced the activation of T cells and the levels of proinflammatory cytokines and improved cardiac function but not insulin sensitivity.

**Conclusions:** Results provide proof-of-concept evidence for the activation of BM adaptive immunity in T2D. In mice, treatment with abatacept dampens the activation of adaptive immunity and protects from cardiac damage.

## Research in Context

### What Is Already known?

- Type 2 diabetes (T2D) is characterized by systemic low-grade inflammation and activation of innate and adaptive immunity, which supports the novel medical theory that T2D is an autoimmune disease. The bone marrow (BM) is a primary immune organ. People with T2D have a fatty BM characterized by inflamed adipocytes that accumulate at the expense of hematopoietic cells and vascular cells.
- Abatacept, an immunomodulatory drug that acts through the selective inhibition of T-cell co-stimulation, was approved for rheumatoid arthritis in 2007. The drug has shown favorable action in preserving beta cell function in patients with rheumatoid arthritis, possibly due to its anti-inflammatory and immunomodulatory properties.

### What Is the Key Question?

- Is adaptive immunity activated in the BM of patients with T2D?
- Do mice with T2D model the condition seen in patients?
- If so, can abatacept treatment rescue T-cell activation, thereby benefiting metabolic control and cardiac function?

### What Are the New Findings?

- Patients and mice with T2D show a shift in the prevalent subsets of BM T lymphocytes, with enrichment of activated cells.
- Treatment of obese, T2D mice with abatacept rescued the altered adaptive immune profile and improved heart performance, without improving insulin sensitivity.

### How Might This Impact Clinical Practice in the Foreseeable Future?

- The BM requires clinical attention as a target and trigger of inflammatory responses involving cells of the adaptive immunity.
- Multicenter trials are warranted to extend the implication of the study to the general population affected by T2D, to determine if those with an activation of adaptive immunity develop more cardiovascular complications.
- Modern immunomodulatory drugs that disable just the initial stage of lymphocyte activation leaving the remaining immune army unscathed may represent a novel approach to prevent cardiovascular complications.

## Background

Chronic low-grade inflammation plays an important role in the progression of type 2 diabetes (T2D) mellitus and in the increased propensity of diabetic people to develop cardiovascular disease, infections, and cancer (1–4). Both innate and adaptive immunity play key roles in the development of metabolic inflammation and insulin resistance (5–11). Cross-sectional results from the Multi-Ethnic Study of Atherosclerosis (MESA) highlighted the association of T2D with adaptive immune response, reflected by higher memory and lower naive CD4^+^ cells (12). Moreover, higher levels of CD4^+^ memory cells have been reported in the peripheral blood of T2D subjects with cardiovascular disease as compared with those without, suggesting a state of exacerbated immune activation may be implicated in the development of complications (13). Likewise, T lymphocytes infiltrate the visceral adipose tissue (VAT), where they play pivotal roles in the regulation of mechanisms at the intersection of metabolism and immune tolerance (14–17). However, scanty information exists on the T-cell activation in the bone marrow (BM), a tissue crucial for the production and maturation of immune cells.

The BM not only functions as a primary B lymphocyte-producing organ but also represents a site where immune cells are formed, mature, and/or recirculate. Furthermore, the BM plays a key role as a secondary lymphoid organ, crucial for CD4 and CD8 cell-related responses, as well as a site of preferential homing and persistence for memory T cells (18, 19). These comprise subsets with important and specific functionalities, such as T cells, B cells, dendritic cells (DCs), and macrophages. In the BM, memory T cells that maintain a state of readiness after tissue injury can rapidly expand and mount a robust secondary cytotoxic response in case of new challenges, more potently than that afforded by other lymphoid and non-lymphoid organs (20, 21). T cells can home into the BM from the circulation and persist within this organ contributing to systemic immune memory (22). Moreover, the BM contains central CD4 Foxp3 Tregs, which not only suppress the effector T cells but also exert non-immune actions, such as vascular protection, metabolic homeostasis, and tissue repair (23, 24). Immune regulation occurs in the BM through direct cell–cell contacts and soluble factors, including cytokines and chemokines.

Several studies have shown that people with T2D incur a remarkable remodeling of BM, consisting of an accumulation of inflamed adipocytes and depletion of hematopoietic, neuronal, and vascular cells (25–28). These changes impinge on hematopoietic cell functions and contribute to altering the profile and mobilization capacity of hematopoietic stem/progenitor cells (HSPCs) (29, 30). An altered phenotype of released HSPCs reportedly conveys antiangiogenic and proapoptotic features to the peripheral vasculature, thereby contributing to diabetic complications (31). Based on this background, we posit that T2D could also impact BM immune cell homeostasis.

Modern immunomodulatory drugs, which—at variance from classical immunosuppressive agents—disable just a particular type of lymphocyte or interfere with the initial state of lymphocyte activation, leaving the global immune response intact, are attracting much clinical attention. One of these compounds, abatacept, exerts anti-inflammatory activity *via* the selective modulation of T-cell co-stimulation. In Europe, abatacept is approved for use in patients with highly active and progressive rheumatoid arthritis (32). Interestingly, clinical studies in patients with rheumatoid arthritis have shown that abatacept treatment reduced the risk of diabetes and improved markers of metabolic control, through an anti-inflammatory mechanism preserving β-cell function (33–35). However, it remains unknown if abatacept can impact adaptive immune response and preserve target organ damage in diabetes.

The present study was a proof-of-concept investigation assessing the frequencies and activation state of immune cell subsets in the BM of patients with T2D compared with non-diabetic (ND) individuals, with a focus on activated and memory T cells. After confirming human results in a murine model of T2D, we performed a therapeutic preclinical study in a murine model of T2D to determine the effect of abatacept on adaptive immunity, cytokine secretion, metabolic control, and cardiac function.

## Research Design and Methods

### Human Studies

Patients undergoing hip replacement surgery were recruited under informed consent at the Avon Orthopedic Centre, Southmead Hospital, Bristol, UK. The study protocol complied with the Declaration of Helsinki was covered by institutional ethical approval (REC14/SW/1083 and REC14/WA/1005) and was registered as an observational clinical study in the National Institute for Health Research Clinical Research Network Portfolio, UK Clinical Trials Gateway. Demographic and clinical data of the 24 enrolled subjects are reported in Table 1.

**Table 1:** Characteristics of study subjects.

	**ND (*n* = 12)**	**T2D (*n* = 12)**	***P* =**
Age, years	64.0 ± 3.5	70.8 ± 3.0	N.S.
Male, %	50	66	N.S.
BMI, kg/m^2^	26.6 ± 1.6	33.4 ± 1.9	0.018
HbA1c, mmol/mol	N.D.	53.3 ± 2.7	
Smoking, %	42	50	N.S.
Hypertension, %	66	75	N.S.
Coronary Artery Disease, %	17	66	0.01
**Medications**			
Insulin, %	0	42	0.01
Oral anti-diabetic drugs, %	0	66	0.01
Statins, %	25	83	0.01
Anti-hypertensive drugs, %	42	50	N.S.

Type 2 diabetes was diagnosed according to the American Diabetes Association guidelines. Specifically, it was defined as (1) patient/referring doctor reports a previous diagnosis of diabetes, and (2) HbA_1c_ > 48 mmol/mol. We excluded subjects with acute disease/infection, inflammatory/immune diseases, current or past hematological disorders or malignancy, unstable angina, recent (within 6 months) myocardial infarction or stroke, heart failure, liver failure, dialysis, and pregnancy.

#### Human Bone Marrow Collection and Processing

Bone marrow samples were obtained from scooped femur heads remaining from hip replacement surgery. Only material that would otherwise be discarded was collected for the study. During the replacement procedure, the femoral head was removed with a saw, and the proximal femoral canal was opened with reamers and rasps. The BM displaced into the wound was scooped into a sterile pot with a curette. The sample was decanted into a collection tube with 0.5 mol/L ethylenediaminetetraacetic acid (EDTA), pH 8 (Thermo Fisher Scientific, Gloucester, UK, #28348) labeled, and placed in a fridge for collection by the investigator within 1 h.

The diluted BM suspension was passed through a 100-μm filter to remove bone fragments and cell clumps. The sample was stratified on Ficoll Histopaque 1077 (Sigma-Aldrich, St. Louis, MO, USA, #10771) and centrifuged without acceleration or brake at 300 × g for 45 min at 24°C. Mononuclear cells sedimented at the interphase were then collected, washed twice with phosphate buffered saline (PBS), assessed for viability by trypan blue staining (Thermo Fisher Scientific, #15250061), and counted at microscopy. Plasma was separated from freshly collected human BM mononuclear cells (BMMCs) through centrifugation at 3,500 rpm for 10 min and frozen immediately at −80°C.

#### Flow Cytometry Analysis of Human Bone Marrow Samples

The following monoclonal antibody combinations were used to characterize the phenotype of different immune cell subsets: anti-CD45RO-BB515 (#564529), anti-CD3-PE-cy5.5 (#340949), anti-CD4-PE-cy5.5 (#35-0047-42), anti-CD8-PE-cy5 (#15-0088-42), anti-CD45RA-BV605 (#562886), anti-CD25-PE-cy7 (#557741), anti-CD69-APC (#310910), anti-CCR7-Aleza700 (#565867), anti-CD45 BV771 (#304050), anti-CD56PE-cy7 (#557747), anti-CD8-BV510 (#563256), anti-CD19-APC (#340722), anti-CD4BV421 (#565997), anti-CD3PE #340662), and anti-CD19BV785 (#363028). All antibodies were titrated for optimal staining performance.

Cell viability was determined using Zombie NIR™ Fixable Viability Kit (BioLegend, San Diego, CA, USA; #423106). Briefly, 70 × 10^6^ BMMCs (at a concentration of 1 × 10^6^ cells/ml) were stained with Zombie NIR™ Fixable Viability Kit for 30 min at room temperature (RT). Then, the cells were washed with Flow Cytometry Staining Buffer (FACS buffer) (eBioscience™ Flow Cytometry Staining Buffer, eBioscience, Inc., San Diego, CA, USA; #00-4222-57) at 300 × g for 10 min. The staining protocol included the use of an Fc blocking antibody (True-Stain Monocyte Blocker, BioLegend, #426102) to control for non-specific binding and background fluorescence. Each sample was added with 100 μl Fc blocking antibody (diluted in FACS buffer at 1:50 ratio) and incubated on ice for 20 min. The cells were then centrifuged at 500 × g for 5 min at 4°C. Subsequently, BMMCs were stained with a defined combination of antibodies, at the appropriate dilutions, in FACS buffer for 20 min at 4°C. Cells were washed twice in FACS buffer and stained with 1% PFA. A minimum of 2 × 10^6^ cells/ml was assayed in the flow cytometry studies. Fluorescence minus one (FMO) controls were included in building multicolor flow cytometry panels. Moreover, BD Comp Beads (#560497) were used to optimize fluorescence compensation settings in multicolor flow cytometry studies. The analysis was performed on a BD LSR II Fortessa X20 (Becton Dickinson, San Jose, CA, USA) using FlowJo software (TreeStar, San Carlos, CA, USA).

### Animal Studies

Experiments were performed following the Guide for the Care and Use of Laboratory Animals (The Institute of Laboratory Animal Resources, 1996) and with the approval of the University of Bristol and the British Home Office (license 30/3373). As a model of T2D, we used a 10-week-old male obese leptin receptor homozygous mutant BKS.Cg-+Leprdb/+Leprdb/OlaHsd (db/db) mice (Envigo, Blackthorn, UK). Age- and sex-matched lean heterozygous + BKS.Cg-+Leprdb/+LeprWT/OlaHsd (db/WT) mice served as controls. Animals were maintained in the high standard animal facilities of the University of Bristol, under the surveillance of specialists trained in husbandry and welfare of the species. The animals fed standard chow (EURodent Diet 22%, www.LabDiet.com) and provided water *ad libitum*. Glucose was measured in urine using Diastix colorimetric reagent strips (Bayer, Reading, UK). At sacrifice, the BM, spleen, and peripheral blood were collected according to the procedures described below.

#### Abatacept Treatment

Abatacept (250 mg lyophilized powder per vial, Bristol-Myers Squibb, Princeton, NJ, USA) was purchased from the Bristol University Hospital Pharmacy. The drug was reconstituted at 2× of the concentration required in sterile distilled water (25 mg/ml). The vial was gently swirled until the complete dissolution of the compound. The reconstituted material was diluted with sterile pH-checked PBS for *in vivo* use on the same day of preparation.

A cohort of twelve 10-week-old male db/db mice was randomized for treatment with abatacept or vehicle. Mice were injected intraperitoneally with 300 μg abatacept in 100 μl PBS, three non-consecutive times a week, for 4 weeks, while controls received the vehicle. The dosage and control choice were adapted from a previous abatacept study in a murine model of cardiomyopathy (36). Dimensional and functional parameters of the heart were measured before sacrifice using a Vevo3100 echocardiography system, using an MX400 transducer (Fujifilm VisualSonics Inc, Toronto, ON, Canada). The echocardiography study was performed with mice under isoflurane anesthesia (2.5% for induction, followed by 0.5–1.2% as appropriate to maintain the heart rate between 400 and 450 bpm).

#### Collection and Processing of Bone Marrow

At sacrifice, whole BM cells were obtained from the mouse tibiae and femora. Both ends of the bones were cut using sterile, sharpened scissors. BM cells were harvested by repeated flushing of the bone shaft in 1 ml PBS in a tube using a syringe provided with a 23-gauge needle. After removal of aggregates from the BM suspension by vigorous pipetting and filtration through a 70-μm mesh nylon strainer (#07-201-431, Thermo Fisher Scientific), the sample was centrifuged at 300 × g for 10 min. The pellets were resuspended to obtain a suspension of 2 × 10^6^ cells/ml in fresh PBS for use in cytometry analysis. Supernatants were collected for cytokine assays and stored at −20°C.

#### Collection and Processing of Splenocytes

Mononuclear spleen suspensions were prepared by gently pressing the spleen tissue with the flat end of a syringe in 5 ml of Roswell Park Memorial Institute Medium (RPMI) containing 1% pen/strep, on a 100-mm culture dish. Then, the cell suspension was passed through on a 70-μm cell strainer into a 50-ml tube. After washing twice with 5-ml RPMI, the cell suspension was centrifuged at 300 × g for 10 min, and cells were resuspended in eBioscience™ 1 × red blood cell (RBC) Lysis Buffer (#00-4333-57) on ice for 3 min. Finally, splenocytes were washed, resuspended in PBS at a concentration of 2 × 10^6^ cells/ml, and counted.

#### Collection and Processing of Peripheral Blood

At sacrifice, the blood was harvested with an EDTA-coated syringe from the left ventricle and placed into Eppendorf tubes (Eppendorf, Hamburg, Germany). Blood was centrifuged for 15 min at 3,000 rpm (1,500 × g) at 4°C. After centrifugation, the plasma was removed, and the pellet was incubated with 1× RBC Lysis Buffer on ice for 10 min. Mononuclear cells were then resuspended at 2 × 10^6^ cells/ml in PBS. Plasma was stored at −80°C for later analysis.

#### Flow Cytometry Analyses

Immunofluorescence surface staining was performed by adding a panel of directly conjugated mAb to freshly prepared BMMCs, splenocytes, and peripheral blood mononuclear cells. Before staining, cell viability was assessed using Zombie NIR™ Fixable Viability Kit (#423106) for 30 min at RT. For all experiments, cell suspensions were preincubated with anti-CD16/CD32 mAb (BioLegend) to block FcγRII/III receptors. Cell staining was performed in the dark for 20 min at 4°C in FACS staining buffer (BD, #554656). The following reagents were used: CD4-PE-cy5.5 (#35-0042-82), CD8-PE-cy5 (#15-0083-81), CD25-FiTC (#130-120-172), CD45-BV785 (#103149), CD69 PE-Vio®770 (#30-103-944), NK1.1-AF700 (#108729), CD19-BV510 (#115545), CD44-Super Bright (#63-0441-82), CD62l BV650 (#564108), F4/80-APC (#123115), CD206-PE (#141705), CD11b-PE-cy 7 (#101215), CD80-PEdazzle (#104737), CD11c-BV605 (#117333), and MHCII-AF700 (#107621). The proliferation of CD4^+^ and CD8^+^ cells was assessed using the KI67-BV510 marker (#563462). Cells were fixed and permeabilized using the BD Pharmingen™ Transcription Factor Buffer Set Kit (#562574; BD Pharmingen Inc., San Diego, CA, USA). A minimum of 2 × 10^6^ cells/ml was assayed in the flow cytometry studies. BD Anti-Mouse Ig, κ/Negative Control (BSA) Compensation Plus (7.5 μm) Particles Set (#560497) were used to optimize fluorescence compensation settings in multicolor flow cytometry analysis. Cells were analyzed on LSR II Fortessa X20 Flow Cytometry instrument using the FlowJo software.

#### Cytokine Profiling

Cytokine content in plasma separated from BM was measured using the Proteome Profiler Human Cytokine Array Kit (Research & Diagnostic Systems, Inc, Minneapolis, MN, USA; #ARY005B). A dedicated mouse Cytokine Array Kit (#ARY006) was used to measure cytokines in the BM supernatants and the plasma isolated from the peripheral blood. The cytokines were detected by exposing the membrane to X-ray film, which was subsequently developed with ChemiDoc XRS+ System by Bio-Rad (Hercules, California, USA). The mean luminescence was normalized to reference spots from the same membrane following background correction. Pixel densities on developed X-ray film were analyzed using Fiji—ImageJ image analysis software (National Institutes of Health, Bethesda, MD, USA).

#### Insulin and Glucose Assays

Plasma levels of insulin and glucose were measured using colorimetric assay kits (from Thermo Fisher Scientific, #EMINS, and Abcam, #ab65333, respectively). The quantitative insulin sensitivity check index (QUICKI) was calculated according to the formula QUICKI = 1/(logI0 + logG0), where I is insulin (μU/ml) and G is glucose (nmol/μl). In addition, the HOMA-IR formula was calculated as fasting insulin multiplied by fasting glucose divided by 22.5.

### Statistical Analysis

Values are presented as mean ± SEM. Two-tailed independent-samples *t*-test was used to compare groups with T2D and without diabetes. Value of *p* < 0.05 was considered statistically significant.

## Results

### T2D Increases the Frequency of T Lymphocytes in Human BM

The two studied groups were similar regarding age and sex distribution (Table 1). The average body mass index (BMI) was 26.6 ± 1.6 in ND subjects and 33.4 ± 1.9 in patients with T2D, thus classifying them as overweight and obese, respectively. Coronary artery disease was 3.9-fold more frequent in patients with T2D. In addition, the assessment of HbA1c levels (53.3 ± 2.7 mmol/mol) indicated that the patients with T2D had an acceptable metabolic control with insulin (42%) and/or oral anti-diabetic drugs (66%).

**Figure 1:**
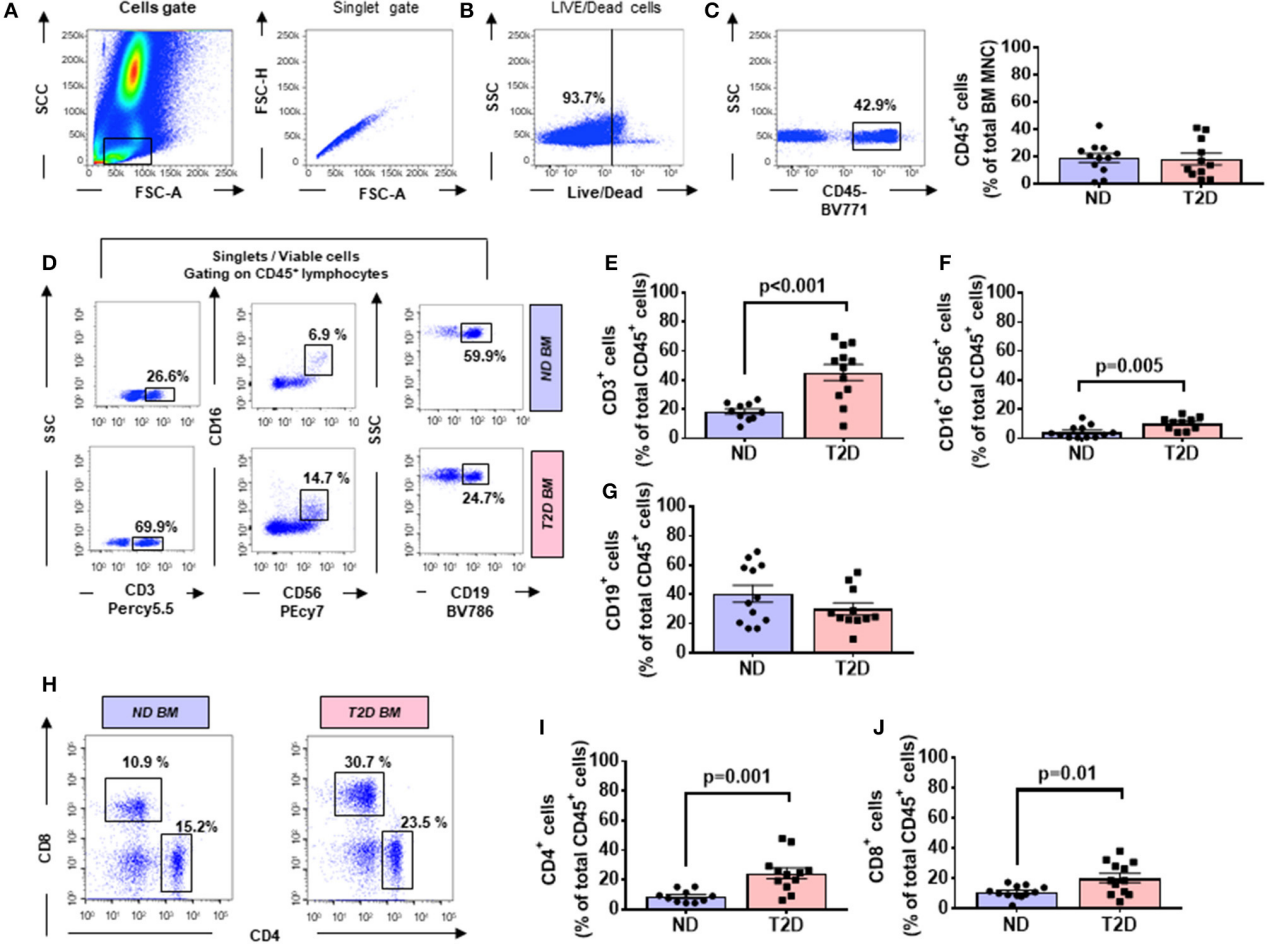
Flow cytometry analysis of immune cells in human bone marrow shows type 2 diabetes (T2D) is associated with an increased abundance of CD4^+^ and CD8^+^ cells. Scooped bone marrow (BM) from femoral head leftovers of orthopedic surgery was obtained from patients previously diagnosed to have T2D and controls without T2D (ND). **(A)** Lymphocytes were gated based on SSC-A vs. FSC-A, and singlets were selected from the FSC-A vs. FSC-H dot plot. **(B)** Subsequently, dead cells were excluded with Zombie NIR™ Fixable Viability Kit. **(C–G)** Lymphocytes were gated based on SSC-A vs. FSC-A, and singlets were selected from CD45^+^ cells to identify population subsets according to the staining for CD3 (T lymphocytes), CD19 (B lymphocytes), and CD16/CD56 (NK cells). **(H–J)** Gating for CD4 and CD8 **(H)** Bar graphs showing the frequency of CD4^+^ cells **(I)** CD8^+^ cells **(J)**. Reported frequencies are illustrative of a representative case for each group. Values are mean ± SEM, with each point representing an individual case.

The flow cytometry analysis of BM cells was performed on data of the CD45/SSC gating, where lymphocytes showed the highest CD45 fluorescence intensity and the lowest SSC signal (Figures 1A–C). As indicated in section Research Design and Methods, the FMO control was used as a control in all the experiments. Cell viability was consistently >90% (Figure 1B). There were no differences in the total cellularity per ml of sample (data not shown) and the frequency of CD45 cells between T2D and ND groups (Figure 1C, *p* = 0.88). We next analyzed lymphocytes, namely, T cells (CD45^+^CD3^+^), natural killer (NK) cells (CD45^+^CD16^+^CD56^+^), and B cells (CD45^+^CD19^+^) using the gating strategy shown in Figure 1D. This characterization demonstrated an increase in the frequency of T cells (2.47-fold, *p* < 0.001) and NK cells (2.36-fold, *p* = 0.005) in T2D compared with ND, whereas B cells did not differ between the two groups (*p* = 0.17) (Figures 1E–G). T lymphocytes were further subdivided into CD4^+^ and CD8^+^ cells. Results indicated an increased frequency of both CD4^+^ (2.77-fold, *p* = 0.001) and CD8^+^ T cells (1.84-fold, *p* = 0.01) in T2D compared with ND (Figures 1H–J). Values are mean, with single points indicating individual samples.

### T2D Increases the Frequency of Activated T Lymphocytes

**Figure 2:**
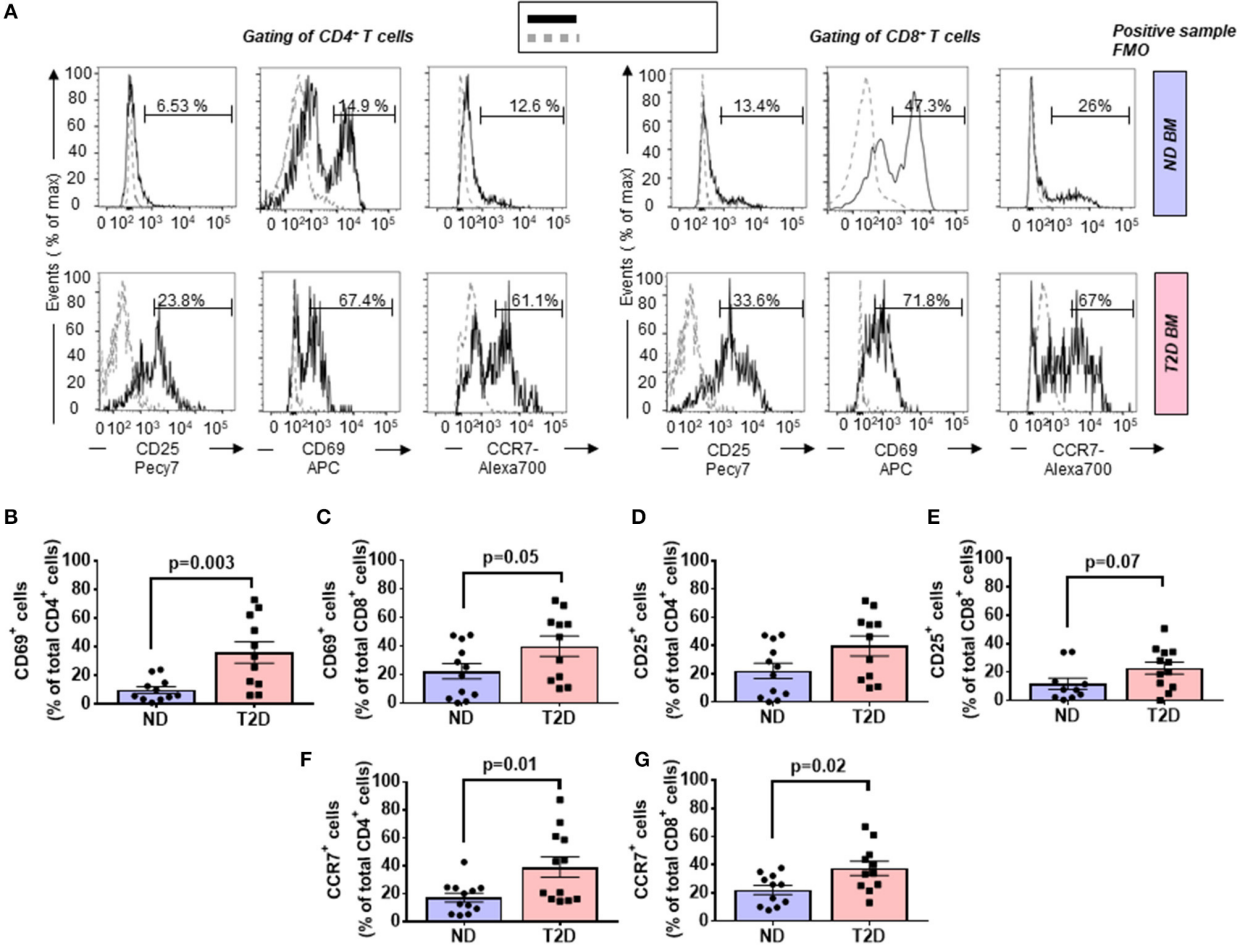
Increased relative abundance of activated T cells in the bone marrow (BM) of patients with T2D. **(A)** Representative histogram overlays of CD4^+^ (left panels) and CD8^+^ cells (right panels) expressing the activation markers CD25 (late marker), CD69 (early marker), and chemokine receptor CCR7 (black line histogram) and FMO control (gray dotted histogram). BM-MCs were stained as described in Materials and Methods with mAb mixture containing BV771-CD45, PE-cy5-CD8, PE-cy5.5-CD4, PE-cy7-CD25, APC-CD69, and Alexa700-CCR7. Reported frequencies are illustrative of a representative case for each group. **(B,C)** Relative frequency of CD69 within CD4^+^ and CD8^+^ cells. **(D,E)** Relative frequency of CD25 within CD4^+^ and CD8^+^ cells. **(F,G)** Relative frequency of CCR7 within CD4^+^ and CD8^+^ cells. Values are mean ± SEM, with each point representing an individual case.

T cells are activated through the presentation of antigens expressed on the surface of antigen-presenting cells (APCs). Once activated, they divide rapidly and secrete cytokines that regulate and sustain the immune response. Previous studies have shown that the human BM contains T cells in the activated state as denoted by the expression of CD69 (37). Here, we report that BM CD4^+^ and CD8^+^ T cells from patients with T2D have a heightened activation state compared with ND individuals, as indicated by an increased frequency of CD69 (Figure 2A). The highest expression of CD69 was observed in the CD4^+^ fraction, which was increased 3.64-fold in T2D (*p* = 0.003 vs. ND), while the increase was 1.79-fold for the CD8^+^ fraction (*p* = 0.05 vs. ND) (Figures 2B,C). We looked for confirmation of T-cell activation by checking the expression of the late marker CD25. Data indicate a more modest difference between the two groups, not reaching statistical significance (*p* = 0.15 and 0.07, for CD4^+^ and CD8^+^ cells, respectively) (Figures 2D,E).

### Type 2 Diabetes Increases the Frequency of CCR7-Expressing T Lymphocytes

**Figure 3:**
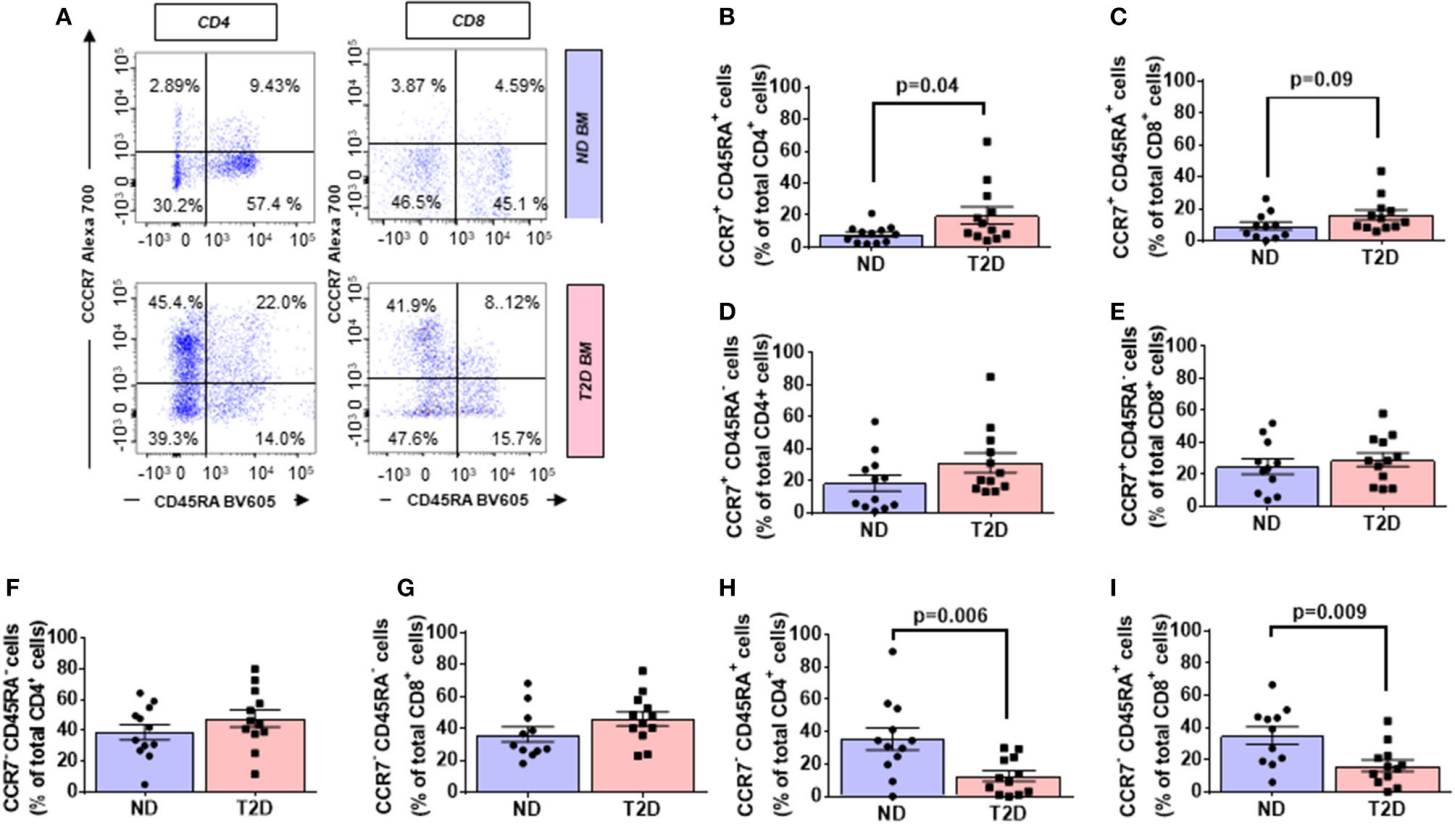
Increased relative abundance of CCR7 expressing T lymphocytes in BM of patients with T2D. **(A)** Gating strategy for identification of CCR7 and CD45RA. The numbers indicate the percentage of naïve T cells (CCR7^+^ CD45RA^+^, top right quadrant), central memory T cells (TCM, CCR7^+^ CD45RA^−^, top left quadrant), effector memory T cells (TEM, CCR7^−^ CD45RA^−^, bottom left quadrant), and terminal effector T cells (TEMRA, CCR7^−^ CD45RA^+^, bottom right quadrant) gated on the forward and side scatter of the lymphocyte populations. Reported frequencies are illustrative of a representative case for each group. **(B–I)** Bar graphs showing the relative frequency of CCR7^+^ CD45RA^+^ naive cells in CD4^+^ **(B)** CD8^+^ cells **(C)** CCR7^+^ CD45RA^−^ TCM cells in CD4^+^ **(D)** and CD8^+^ cells **(E)**, CCR7^−^ CD45RA^−^ TEM cells in CD4^+^ **(F)** and CD8^+^ cells **(G)** CCR7^−^CD45RA^+^ TEMRA cells in CD4^+^ **(H)** and CD8^+^ cells **(I)**. Values are mean ± SEM, with each point representing an individual case.

The CC-chemokine receptor 7 (CCR7) and its ligands play a key role in lymphocyte homing to lymphoid tissue. In the T2D group, both CD4^+^ and CD8^+^ lymphocytes expressed CCR7 with higher frequency than the ND group (2.27-fold, *p* = 0.01; and 1.69-fold, *p* = 0.02, respectively) (gating is shown in Figure 2A and results in Figures 2F,G). Moreover, we combined CCR7 and CD45RA antigens in the flow cytometry analysis to distinguish naïve and memory subpopulations (gating strategy is shown in Figure 3A). Naive CCR7^+^CD45RA^+^ T cells were more abundant in T2D, with 19.9 ± 5.4% of the CD4^+^ cell subfraction expressing this phenotype (2.46-fold more than ND, *p* = 0.04) (Figure 3B). Within CD8^+^ cells, naive T lymphocytes averaged 16.2 ± 3.2% (1.76-fold more than ND, *p* = 0.09) (Figure 3C). Naive lymphocytes are patrolling precursor cells that travel in and out of lymphoid organs in search of cognate antigens. Hence, changes in the local microenvironment might have contributed to the recruitment and homing of naive cells to the BM. Here, APCs could induce the differentiation into immune cells capable of effector responses. To test this possibility, we next investigated the influence of T2D on central memory (TCM, CCR7^+^ CD45RA^−^), effector memory (TEM, CCR7^−^ CD45RA), and “revertant” terminally differentiated T memory cells (TEMRA, CCR7^−^ CD45RA^+^). TCM and TEM cells were similar between groups (Figures 3D–G), whereas T2D was characterized by a reduction in the frequency of CD4^+^ and CD8^+^ TEMRA (0.34-fold, *p* = 0.006; and 0.47-fold vs. ND, *p* = 0.009, respectively) (Figures 3H,I).

A multivariable regression model including age, sex, and BMI as covariates confirmed the differences emerging from the univariate analysis illustrated in previous paragraphs.

### Altered Chemokine Profile in the BM of Patients With T2D

**Figure 4:**
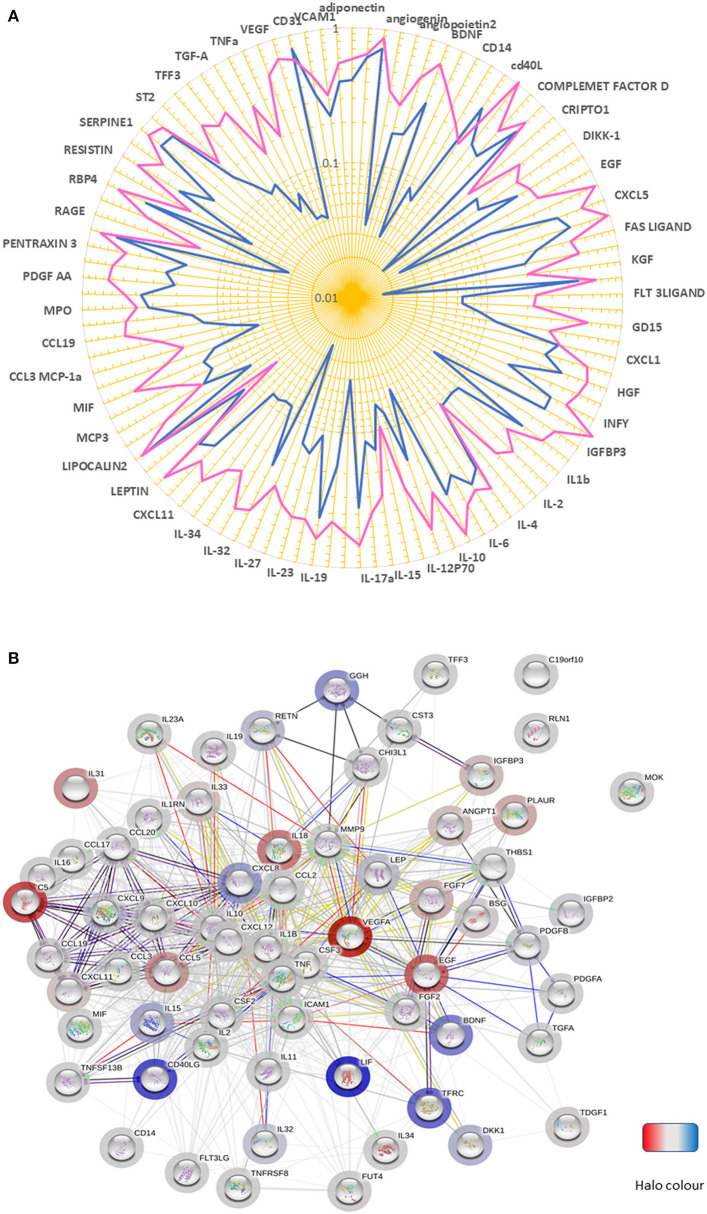
Altered chemokine profile in BM of patients with T2D. **(A)** Spider graph showing the expression levels of chemokines, cytokines, and proteins measured using a proteome profiling array in bone marrow perfusates of three subjects per group. Pink line (T2D) and the blue line (ND). **(B)** The network of factors found to be differentially regulated between T2D and ND. The bar halo color is based on the rank of the protein in the set of input values. Action types are shown as: 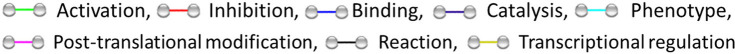.

We next examined the expression of a panel of cytokines, chemokines, and proteins in BM perfusates from three patients with T2D and three ND subjects. Of 104 measured factors, 65 were upregulated and 2 downregulated in T2D BM, considering a threshold fold change ≥2 (Figure 4A, Table 2).

**Table 2:** List of modulated factors in human bone marrow with value of the fold change vs. ND.

**Node**	**Annotation**	**Fold change**	***p*-value**
CD40LG	CD40 ligand; Cytokine that binds to CD40/TNFRSF5. Costimulates T-cell proliferation and cytokine production. Its cross-linking on T-cells generates a costimulatory signal which enhances the production of IL4 and IL10 in conjunction with the TCR/CD3 ligation and CD28 costimulation. Induces the activation of NF-kappa-B and kinases MAPK8 and PAK2 in T- cells. Induces tyrosine phosphorylation of isoform 3 of CD28. Mediates B-cell proliferation in the absence of co-stimulus as well as IgE production in the presence of IL4. Involved in immunoglobulin class switching (By similarity)	0.48	0.41
LIF	Leukemia inhibitory factor; LIF has the capacity to induce terminal differentiation in leukemic cells. Its activities include the induction of hematopoietic differentiation in normal and myeloid leukemia cells, the induction of neuronal cell differentiation, and the stimulation of acute-phase protein synthesis in hepatocytes; Endogenous ligands	0.48	0.85
BDNF	Brain-derived neurotrophic factor; During development, promotes the survival and differentiation of selected neuronal populations of the peripheral and central nervous systems. Participates in axonal growth, pathfinding and in the modulation of dendritic growth and morphology. Major regulator of synaptic transmission and plasticity at adult synapses in many regions of the CNS	2.01	0.24
GGH	Gamma-glutamyl hydrolase; Hydrolyzes the polyglutamate sidechains of pteroylpolyglutamates. Progressively removes gamma-glutamyl residues from pteroylpoly-gamma-glutamate to yield pteroyl-alpha- glutamate (folic acid) and free glutamate. May play an important role in the bioavailability of dietary pteroylpolyglutamates and in the metabolism of pteroylpolyglutamates and antifolates; Belongs to the peptidase C26 family	2.10	0.27
CXCL8	Interleukin-8; IL-8 is a chemotactic factor that attracts neutrophils, basophils, and T-cells, but not monocytes. It is also involved in neutrophil activation. It is released from several cell types in response to an inflammatory stimulus. IL-8(6-77) has a 5-10-fold higher activity on neutrophil activation, IL-8(5-77) has increased activity on neutrophil activation and IL-8(7-77) has a higher affinity to receptors CXCR1 and CXCR2 as compared to IL-8(1-77), respectively; Chemokine ligands	2.13	0.13
IL15	Interleukin-15; Cytokine that stimulates the proliferation of T- lymphocytes. Stimulation by IL-15 requires interaction of IL-15 with components of IL-2R, including IL-2R beta and probably IL-2R gamma but not IL-2R alpha; Belongs to the IL-15/IL-21 family	2.20	0.59
DKK1	Dickkopf-related protein 1; Antagonizes canonical Wnt signaling by inhibiting LRP5/6 interaction with Wnt and by forming a ternary complex with the transmembrane protein KREMEN that promotes internalization of LRP5/6	2.23	0.58
LEP	Leptin; Key player in the regulation of energy balance and body weight control. Once released into the circulation, has central and peripheral effects by binding LEPR, found in many tissues, which results in the activation of several major signaling pathways.	2.30	0.13
RETN	Resistin; Hormone that seems to suppress insulin ability to stimulate glucose uptake into adipose cells (By similarity). Potentially links obesity to diabetes (By similarity). Promotes chemotaxis in myeloid cells	2.30	0.10
IL32	Interleukin-32; Interleukin 32; Interleukins	2.40	0.31
CXCL9	C-X-C motif chemokine 9; Cytokine that affects the growth, movement, or activation state of cells that participate in immune and inflammatory response. Chemotactic for activated T-cells. Binds to CXCR3; Belongs to the intercrine alpha (chemokine CxC) family	2.49	0.59
IGFBP2	Insulin-like growth factor-binding protein 2; Inhibits IGF-mediated growth and developmental rates. IGF-binding proteins prolong the half-life of the IGFs and have been shown to either inhibit or stimulate the growth promoting effects of the IGFs on cell culture. They alter the interaction of IGFs with their cell surface receptors	2.50	0.008
PDGFB	Platelet-derived growth factor subunit B; Growth factor that plays an essential role in the regulation of embryonic development, cell proliferation, cell migration, survival and chemotaxis. Potent mitogen for cells of mesenchymal origin. Required for normal proliferation and recruitment of pericytes and vascular smooth muscle cells in the central nervous system, skin, lung, heart and placenta. Required for normal blood vessel development, and for normal development of kidney glomeruli	2.50	0.15
CSF2	Granulocyte-macrophage colony-stimulating factor; Cytokine that stimulates the growth and differentiation of hematopoietic precursor cells from various lineages, including granulocytes, macrophages, eosinophils and erythrocytes; Belongs to the GM-CSF family	2.52	0.55
CST3	Cystatin-C; As an inhibitor of cysteine proteinases, this protein is thought to serve an important physiological role as a local regulator of this enzyme activity; Belongs to the cystatin family	2.55	0.45
FGF2	Fibroblast growth factor 2; Plays an important role in the regulation of cell survival, cell division, angiogenesis, cell differentiation and cell migration. Functions as potent mitogen *in vitro*. Can induce angiogenesis; Belongs to the heparin-binding growth factors family	2.55	0.32
IL19	Interleukin-19; May play some important roles in inflammatory responses. Up-regulates IL-6 and TNF-alpha and induces apoptosis (By similarity); Interleukins	2.60	0.36
TNFSF13B	Tumor necrosis factor ligand superfamily member 13B; Cytokine that binds to TNFRSF13B/TACI and TNFRSF17/BCMA. TNFSF13/APRIL binds to the same 2 receptors. Together, they form a 2 ligands −2 receptors pathway involved in the stimulation of B- and T-cell function and the regulation of humoral immunity. A third B-cell specific BAFF-receptor (BAFFR/BR3) promotes the survival of mature B-cells and the B-cell response; CD molecules	2.71	0.31
C19orf10	Myeloid-derived growth factor; Bone marrow-derived monocyte and paracrine-acting protein that promotes cardiac myocyte survival and adaptive angiogenesis for cardiac protection and/or repair after myocardial infarction (MI). Stimulates endothelial cell proliferation through a MAPK1/3-, STAT3-, and CCND1-mediated signaling pathway. Inhibits cardiac myocyte apoptosis in a PI3K/AKT-dependent signaling pathway (By similarity). Involved in endothelial cell proliferation and angiogenesis	2.90	0.38
CXCL12	Stromal cell-derived factor 1; Chemoattractant active on T-lymphocytes, monocytes, but not neutrophils. Activates the C-X-C chemokine receptor CXCR4 to induce a rapid and transient rise in the level of intracellular calcium ions and chemotaxis. Also binds to atypical chemokine receptor ACKR3, which activates the beta-arrestin pathway and acts as a scavenger receptor for SDF-1. SDF-1-beta(3-72) and SDF-1- alpha(3-67) show a reduced chemotactic activity	2.90	0.27
IL1B	Interleukin-1 beta; Potent proinflammatory cytokine. Initially discovered as the major endogenous pyrogen, induces prostaglandin synthesis, neutrophil influx and activation, T-cell activation and cytokine production, B-cell activation and antibody production, and fibroblast proliferation and collagen production. Promotes Th17 differentiation of T-cells	2.90	0.51
PDGFA	Platelet-derived growth factor subunit A; Growth factor that plays an essential role in the regulation of embryonic development, cell proliferation, cell migration, survival and chemotaxis. Potent mitogen for cells of mesenchymal origin. Required for normal lung alveolar septum formation during embryogenesis, normal development of the gastrointestinal tract, normal development of Leydig cells and spermatogenesis	3.00	0.09
MMP9	Matrix metalloproteinase-9; May play an essential role in local proteolysis of the extracellular matrix and in leukocyte migration. Could play a role in bone osteoclastic resorption. Cleaves KiSS1 at a Gly-|-Leu bond. Cleaves type IV and type V collagen into large C-terminal three quarter fragments and shorter N-terminal one quarter fragments. Degrades fibronectin but not laminin or Pz-peptide; M10 matrix metallopeptidases	3.10	0.20
RLN1	Prorelaxin H1; Relaxin is an ovarian hormone that acts with estrogen to produce dilatation of the birth canal in many mammals. May be involved in remodeling of connective tissues during pregnancy, promoting growth of pubic ligaments and ripening of the cervix; Belongs to the insulin family	3.10	0.61
TGFA	Protransforming growth factor alpha; TGF alpha is a mitogenic polypeptide that is able to bind to the EGF receptor/EGFR and to act synergistically with TGF beta to promote anchorage-independent cell proliferation in soft agar	3.20	0.69
MOK	MAPK/MAK/MRK overlapping kinase; Able to phosphorylate several exogenous substrates and to undergo autophosphorylation. Negatively regulates cilium length in a cAMP and mTORC1 signaling-dependent manner; Belongs to the protein kinase superfamily. CMGC Ser/Thr protein kinase family. CDC2/CDKX subfamily	3.30	0.73
CSF3	Granulocyte colony-stimulating factor; Granulocyte/macrophage colony-stimulating factors are cytokines that act in hematopoiesis by controlling the production, differentiation, and function of 2 related white cell populations of the blood, the granulocytes and the monocytes-macrophages. This CSF induces granulocytes; Belongs to the IL-6 superfamily	3.40	0.62
IL16	Pro-interleukin-16; Interleukin-16 stimulates a migratory response in CD4+ lymphocytes, monocytes, and eosinophils. Primes CD4+ T-cells for IL-2 and IL-15 responsiveness. Also induces T-lymphocyte expression of interleukin 2 receptor. Ligand for CD4; Interleukins	3.4	0.37
IL2	Interleukin-2; Produced by T-cells in response to antigenic or mitogenic stimulation, this protein is required for T-cell proliferation and other activities crucial to regulation of the immune response. Can stimulate B-cells, monocytes, lymphokine- activated killer cells, natural killer cells, and glioma cells; Interleukins	3.47	0.40
FLT3LG	Fms-related tyrosine kinase 3 ligand; Stimulates the proliferation of early hematopoietic cells by activating FLT3. Synergizes well with a number of other colony stimulating factors and interleukins; Endogenous ligands	3.50	0.60
IL11	Interleukin-11; Cytokine that stimulates the proliferation of hematopoietic stem cells and megakaryocyte progenitor cells and induces megakaryocyte maturation resulting in increased platelet production. Also promotes the proliferation of hepatocytes in response to liver damage. Binding to its receptor formed by IL6ST and either IL11RA1 or IL11RA2 activates a signaling cascade that promotes cell proliferation. Signaling leads to the activation of intracellular protein kinases and the phosphorylation of STAT3; Belongs to the IL-6 superfamily	3.51	0.14
CCL20	C-C motif chemokine 20; Acts as a ligand for C-C chemokine receptor CCR6. Signals through binding and activation of CCR6 and induces a strong chemotactic response and mobilization of intracellular calcium ions. The ligand-receptor pair CCL20-CCR6 is responsible for the chemotaxis of dendritic cells (DC), effector/memory T-cells and B- cells and plays an important role at skin and mucosal surfaces under homeostatic and inflammatory conditions	3.60	0.51
ICAM1	Intercellular adhesion molecule 1; ICAM proteins are ligands for the leukocyte adhesion protein LFA-1 (integrin alpha-L/beta-2). During leukocyte trans- endothelial migration, ICAM1 engagement promotes the assembly of endothelial apical cups through ARHGEF26/SGEF and RHOG activation; CD molecules	3.60	0.06
MIF	Macrophage migration inhibitory factor; Pro-inflammatory cytokine. Involved in the innate immune response to bacterial pathogens. The expression of MIF at sites of inflammation suggests a role as mediator in regulating the function of macrophages in host defense. Counteracts the anti- inflammatory activity of glucocorticoids. Has phenylpyruvate tautomerase and dopachrome tautomerase activity (*in vitro*), but the physiological substrate is not known. It is not clear whether the tautomerase activity has any physiological relevance, and whether it is important for cytokine activity	3.60	0.10
CHI3L1	Chitinase-3-like protein 1; Carbohydrate-binding lectin with a preference for chitin. Has no chitinase activity. May play a role in tissue remodeling and in the capacity of cells to respond to and cope with changes in their environment. Plays a role in T-helper cell type 2 (Th2) inflammatory response and IL-13-induced inflammation, regulating allergen sensitization, inflammatory cell apoptosis, dendritic cell accumulation and M2 macrophage differentiation. Facilitates invasion of pathogenic enteric bacteria into colonic mucosa and lymphoid organs	3.76	0.003
CCL17	C-C motif chemokine 17; Chemotactic factor for T-lymphocytes but not monocytes or granulocytes. May play a role in T-cell development in thymus and in trafficking and activation of mature T-cells. Binds to CCR4; Belongs to the intercrine beta (chemokine CC) family	3.80	0.24
CCL3	C-C motif chemokine 3; Monokine with inflammatory and chemokinetic properties. Binds to CCR1, CCR4, and CCR5. One of the major HIV-suppressive factors produced by CD8+ T-cells. Recombinant MIP-1-alpha induces a dose-dependent inhibition of different strains of HIV-1, HIV-2, and simian immunodeficiency virus (SIV); Belongs to the intercrine beta (chemokine CC) family	3.80	0.63
CCL19	C-C motif chemokine 19; May play a role not only in inflammatory and immunological responses but also in normal lymphocyte recirculation and homing. May play an important role in trafficking of T-cells in thymus, and T-cell and B-cell migration to secondary lymphoid organs. Binds to chemokine receptor CCR7. Recombinant CCL19 shows potent chemotactic activity for T-cells and B-cells but not for granulocytes and monocytes. Binds to atypical chemokine receptor ACKR4 and mediates the recruitment of beta-arrestin (ARRB1/2) to ACKR4; Belongs to the intercrine beta (chemokine CC) family	3.90	0.23
IL34	Interleukin-34; Cytokine that promotes the proliferation, survival and differentiation of monocytes and macrophages. Promotes the release of proinflammatory chemokines, and thereby plays an important role in innate immunity and in inflammatory processes. Plays an important role in the regulation of osteoclast proliferation and differentiation, and in the regulation of bone resorption. Signaling via CSF1R and its downstream effectors stimulates phosphorylation of MAPK1/ERK2 AND MAPK3/ERK1; Belongs to the IL-34 family	4.10	0.42
IL1RN	Interleukin-1 receptor antagonist protein; Inhibits the activity of interleukin-1 by binding to receptor IL1R1 and preventing its association with the coreceptor IL1RAP for signaling. Has no interleukin-1 like activity. Binds functional interleukin-1 receptor IL1R1 with greater affinity than decoy receptor IL1R2; however, the physiological relevance of the latter association is unsure; Endogenous ligands	4.23	0.28
IL23A	Interleukin-23 subunit alpha; Associates with IL12B to form the IL-23 interleukin, a heterodimeric cytokine which functions in innate and adaptive immunity. IL-23 may constitute with IL-17 an acute response to infection in peripheral tissues. IL-23 binds to a heterodimeric receptor complex composed of IL12RB1 and IL23R, activates the Jak- Stat signaling cascade, stimulates memory rather than naive T- cells and promotes production of proinflammatory cytokines. IL-23 induces autoimmune inflammation	4.30	0.24
TFF3	Trefoil factor 3; Involved in the maintenance and repair of the intestinal mucosa. Promotes the mobility of epithelial cells in healing processes (motogen)	4.36	0.21
CXCL10	C-X-C motif chemokine 10; Chemotactic for monocytes and T-lymphocytes. Binds to CXCR3; Belongs to the intercrine alpha (chemokine CxC) family	4.40	0.36
TFRC	Transferrin receptor protein 1; Cellular uptake of iron occurs via receptor-mediated endocytosis of ligand-occupied transferrin receptor into specialized endosomes. Endosomal acidification leads to iron release. The apotransferrin-receptor complex is then recycled to the cell surface with a return to neutral pH and the concomitant loss of affinity of apotransferrin for its receptor. Transferrin receptor is necessary for development of erythrocytes and the nervous system (By similarity)	4.45	0.16
TNFRSF8	Tumor necrosis factor receptor superfamily member 8; Receptor for TNFSF8/CD30L. May play a role in the regulation of cellular growth and transformation of activated lymphoblasts. Regulates gene expression through activation of NF- kappa-B	4.47	0.44
CD14	Monocyte differentiation antigen CD14; Coreceptor for bacterial lipopolysaccharide. In concert with LBP, binds to monomeric lipopolysaccharide and delivers it to the LY96/TLR4 complex, thereby mediating the innate immune response to bacterial lipopolysaccharide (LPS). Acts via MyD88, TIRAP and TRAF6, leading to NF-kappa-B activation, cytokine secretion and the inflammatory response	4.70	0.32
TNF	Tumor necrosis factor; Cytokine that binds to TNFRSF1A/TNFR1 and TNFRSF1B/TNFBR. It is mainly secreted by macrophages and can induce cell death of certain tumor cell lines. It is potent pyrogen causing fever by direct action or by stimulation of interleukin-1 secretion and is implicated in the induction of cachexia, Under certain conditions it can stimulate cell proliferation and induce cell differentiation. Impairs regulatory T-cells (Treg) function in individuals with rheumatoid arthritis via FOXP3 dephosphorylation	4.70	0.60
THBS1	Thrombospondin-1; Adhesive glycoprotein that mediates cell-to-cell and cell-to-matrix interactions. Binds heparin. May play a role in dentinogenesis and/or maintenance of dentin and dental pulp (By similarity). Ligand for CD36 mediating antiangiogenic properties. Plays a role in ER stress response, via its interaction with the activating transcription factor 6 alpha (ATF6) which produces adaptive ER stress response factors (By similarity)	5.30	0.21
CCL2	C-C motif chemokine 2; Chemotactic factor that attracts monocytes and basophils but not neutrophils or eosinophils. Augments monocyte anti-tumor activity. Has been implicated in the pathogenesis of diseases characterized by monocytic infiltrates, like psoriasis, rheumatoid arthritis or atherosclerosis. May be involved in the recruitment of monocytes into the arterial wall during the disease process of atherosclerosis; Belongs to the intercrine beta (chemokine CC) family	5.70	0.23
FUT4	Alpha-(1,3)-fucosyltransferase 4; May catalyze alpha-1,3 glycosidic linkages involved in the expression of Lewis X/SSEA-1 and VIM-2 antigens; CD molecules	6.30	0.59
IL10	Interleukin-10; Inhibits the synthesis of a number of cytokines, including IFN-gamma, IL-2, IL-3, TNF, and GM-CSF produced by activated macrophages and by helper T-cells; Belongs to the IL-10 family	6.69	0.33
TDGF1	Teratocarcinoma-derived growth factor 1; GPI-anchored cell membrane protein involved in Nodal signaling. Cell-associated TDGF1 acts as a Nodal coreceptor in cis. Shedding of TDGF1 by TMEM8A modulates Nodal signaling by allowing soluble TDGF1 to act as a Nodal coreceptor on other cells. Could play a role in the determination of the epiblastic cells that subsequently give rise to the mesoderm	7.04	0.69
BSG	Basigin; Plays an important role in targeting the monocarboxylate transporters SLC16A1, SLC16A3, SLC16A8, and SLC16A11 to the plasma membrane. Plays pivotal roles in spermatogenesis, embryo implantation, neural network formation and tumor progression. Stimulates adjacent fibroblasts to produce matrix metalloproteinases (MMPS). Seems to be a receptor for oligomannosidic glycans. *In vitro*, promotes outgrowth of astrocytic processes; Blood group antigens	7.15	0.01
IL33	Interleukin-33; Cytokine that binds to and signals through the IL1RL1/ST2 receptor which in turn activates NF-kappa-B and MAPK signaling pathways in target cells. Involved in the maturation of Th2 cells inducing the secretion of T-helper type 2-associated cytokines. Also involved in activation of mast cells, basophils, eosinophils and natural killer cells. Acts as a chemoattractant for Th2 cells, and may function as an “alarmin,” that amplifies immune responses during tissue injury; Interleukins	7.30	0.09
CXCL11	C-X-C motif chemokine 11; Chemotactic for interleukin-activated T-cells but not unstimulated T-cells, neutrophils, or monocytes. Induces calcium release in activated T-cells. Binds to CXCR3. May play an important role in CNS diseases which involve T-cell recruitment. May play a role in skin immune responses; Belongs to the intercrine alpha (chemokine CxC) family	8.90	0.12
ANGPT1	Angiopoietin-1; Binds and activates TEK/TIE2 receptor by inducing its dimerization and tyrosine phosphorylation. Plays an important role in the regulation of angiogenesis, endothelial cell survival, proliferation, migration, adhesion and cell spreading, reorganization of the actin cytoskeleton, but also maintenance of vascular quiescence. Required for normal angiogenesis and heart development during embryogenesis. After birth, activates or inhibits angiogenesis, depending on the context	10.12	0.29
IGFBP3	Insulin-like growth factor-binding protein 3; IGF-binding proteins prolong the half-life of the IGFs and have been shown to either inhibit or stimulate the growth promoting effects of the IGFs on cell culture. They alter the interaction of IGFs with their cell surface receptors. Also exhibits IGF-independent antiproliferative and apoptotic effects mediated by its receptor TMEM219/IGFBP-3R	11.7	0.07
FGF7	Fibroblast growth factor 7; Plays an important role in the regulation of embryonic development, cell proliferation and cell differentiation. Required for normal branching morphogenesis. Growth factor active on keratinocytes. Possible major paracrine effector of normal epithelial cell proliferation; Belongs to the heparin-binding growth factors family	12.1	0.54
PLAUR	Urokinase plasminogen activator surface receptor; Acts as a receptor for urokinase plasminogen activator. Plays a role in localizing and promoting plasmin formation. Mediates the proteolysis-independent signal transduction activation effects of U-PA. It is subject to negative-feedback regulation by U-PA which cleaves it into an inactive form; CD molecules	12.9	0.09
CCL5	C-C motif chemokine 5; Chemoattractant for blood monocytes, memory T-helper cells and eosinophils. Causes the release of histamine from basophils and activates eosinophils. May activate several chemokine receptors including CCR1, CCR3, CCR4, and CCR5. One of the major HIV-suppressive factors produced by CD8+ T-cells. Recombinant RANTES protein induces a dose-dependent inhibition of different strains of HIV-1, HIV-2, and simian immunodeficiency virus (SIV).	13.10	0.12
IL31	Interleukin-31; Activates STAT3 and possibly STAT1 and STAT5 through the IL31 heterodimeric receptor composed of IL31RA and OSMR. May function in skin immunity. Enhances myeloid progenitor cell survival *in vitro* (By similarity). Induces RETNLA and serum amyloid A protein expression in macrophages (By similarity); Interleukins	13.3	0.34
IL18	Interleukin-18; Augments natural killer cell activity in spleen cells and stimulates interferon gamma production in T-helper type I cells; Belongs to the IL-1 family	13.6	0.10
EGF	Pro-epidermal growth factor; EGF stimulates the growth of various epidermal and epithelial tissues *in vivo* and *in vitro* and of some fibroblasts in cell culture. Magnesiotropic hormone that stimulates magnesium reabsorption in the renal distal convoluted tubule via engagement of EGFR and activation of the magnesium channel TRPM6. Can induce neurite outgrowth in motoneurons of the pond snail Lymnaea stagnalis *in vitro*	14.4	0.28
C5	Complement C5; Activation of C5 by a C5 convertase initiates the spontaneous assembly of the late complement components, C5-C9, into the membrane attack complex. C5b has a transient binding site for C6. The C5b-C6 complex is the foundation upon which the lytic complex is assembled; C3 and PZP like, alpha-2-macroglobulin domain containing	16.20	0.13
VEGFA	Vascular endothelial growth factor A; Growth factor active in angiogenesis, vasculogenesis and endothelial cell growth. Induces endothelial cell proliferation, promotes cell migration, inhibits apoptosis and induces permeabilization of blood vessels. Binds to the FLT1/VEGFR1 and KDR/VEGFR2 receptors, heparan sulfate and heparin. NRP1/Neuropilin-1 binds isoforms VEGF-165 and VEGF-145. Isoform VEGF165B binds to KDR but does not activate downstream signaling pathways, does not activate angiogenesis and inhibits tumor growth	17.2	0.03

An analysis conducted using the STRING database revealed that most of the differentially regulated factors were connected in a network with a PPI enrichment *p*-value: <1.0e^−16^ (Figure 4B). The highly significant interaction highlights the biological relevance of the phenomenon. Interestingly, in the cytokine series, IL-18, and IL-31 were the most upregulated (both over 13-fold). Among chemokines, CXCL10 and CXCL11 (CXCR3 receptor ligands), MCP-1 (CCR2 ligand), and CCL19 (CCR7 receptor ligand), which are implicated in T-cell migration to sites of inflammation, emerged as the most upregulated factors (all over 3.5-fold). Another ligand for CCR7, CCL21, was not measured because not included in the array, with this representing a limitation of the study. Twenty-six proteins were modulated, including VEGF-A, Complement C5, and EGF (all showing an increase >14-fold). Moreover, basigin (alias CD147), which functions as a receptor for soluble cyclophilins and is involved in cyclophilin-mediated viral infection (38), was upregulated in T2D, together with its transcriptional target MMP-9; whereas LIF, an inhibitor of immune response afforded by T lymphocytes (39), and CD40L, a costimulatory ligand for CD40 involved in modulation of lymphocyte activation (40), were downregulated. Analysis of covariance detected a strong overall effect of T2D on the analyzed factors (*p* < 0.001). Multiple comparison analysis documented a significant difference regarding VEGF-A (*p* = 0.03), basigin (*p* = 0.01), chitinase-3-like protein 1 (*p* = 0.003), and insulin-like growth factor-binding protein 2 (*p* = 0.008).

### Analysis of Murine BM Confirms the Impact of T2D on the Frequency and Activation of T Lymphocytes

**Figure 5:**
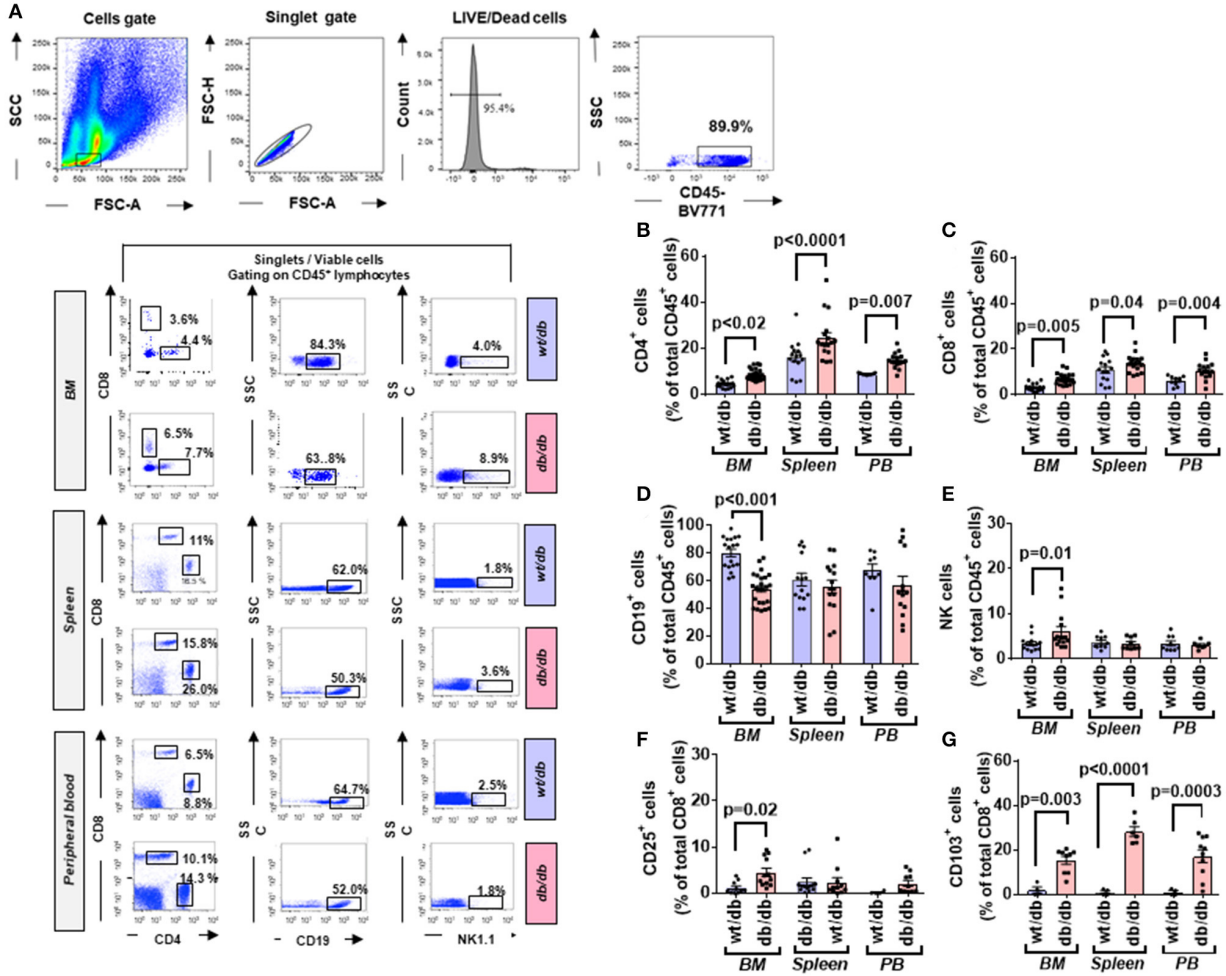
Higher frequency and activated state of T lymphocytes in diabetic mice. **(A)** Gating strategy for identification of CD4, CD8, CD19, and NK1.1 positive cells in the BM, spleen, and peripheral blood of wt/db and db/db mice. Reported frequencies are illustrative of a representative case for each group. **(B)** Relative frequency of CD4^+^ cells. **(C)** Relative frequency of CD8^+^ cells. **(D)** Relative frequency of B cells. **(E)** Relative frequency of NK cells. **(F)** Relative frequency of CD8^+^ cells expressing the activation marker CD25. **(G)** Relative frequency of CD8^+^ cells expressing CD103. Values are mean ± SEM, with each point representing an individual case.

We next sought confirmation about the influence of T2D on immune cells by assessing the phenotype of different cell populations in the BM, spleen, and peripheral blood from 10-week-old db/db and control age-matched wt/db mice (Figure 5A). Confirmation of the diabetic state in the db/db group was achieved by the verification of persistent overt glycosuria. Flow cytometry analysis demonstrated db/db mice had substantial increases in the frequency of CD4^+^ and CD8^+^ cells in the BM (1.88-fold, *p* = 0.02; and 2.23-fold, *p* = 0.005, respectively), spleen (1.53-fold, *p* < 0.0001; 1.27-fold, *p* = 0.04, respectively), and peripheral blood (1.66-fold, *p* = 0.007; and 1.71-fold, *p* = 0.004) (Figures 5B,C), which were associated with decreased abundance of CD19^+^ B cells in the BM (0.67-fold, *p* < 0.0001), but not in the spleen or peripheral blood (Figure 5D). Regarding NK cells, higher numbers were found in BM of db/db mice (1.77-fold, *p* = 0.01), whereas no difference was observed in the other two districts (Figure 5E).

We also extended the analysis beyond lymphocytes to macrophages, identified as CD11b^+^F4/80^+^ cells; they were more abundant in db/db mice at the level of BM (9.25 ± 0.89 vs. 5.77 ± 0.58% in wt/db, *p* = 0.04) and peripheral blood (15.25 ± 3.02 vs. 8.17 ± 1.64% in wt/db, *p* = 0.003), but not in the spleen (4.25 ± 0.76 vs. 2.02 ± 0.40% in wt/db, *p* = 0.56). The M1 subtype, represented by CD80^+^, showed a tendency to increase in the examined sites, albeit not statistically significant (BM: 8.15 ± 1.25 vs. 4.96 ± 1.12% in wt/db, *p* = 0.07; spleen: 3.91 ± 0.46 vs. 2.53 ± 0.46% in wt/db, *p* = 0.07; peripheral blood 7.68 ± 2.07 vs. 3.96 ± 1.16% in wt/db, *p* = 0.18). No difference was observed between groups for CD206^+^ M2 macrophages or MHCII^+^CD11c^+^CD11b^−^CD123^−^ DC (data not shown).

Next, we assessed the specific subsets of CD4^+^ and CD8^+^ cells using immunostaining for the marker of activation CD25. No difference between db/db and wt/db mice was observed regarding CD4^+^ cells (data not shown). In contrast, as shown in Figure 5F, higher levels of CD8^+^ cells stained positive for CD25 in db/db mice at the level of the BM (3.87-fold, *p* = 0.02) but not in the spleen or peripheral blood. Finally, we determined the expression of CD103, an integrin that identifies tissue-resident-memory CD8^+^ cells with an immunosurveillance and protective function (41) and is dynamically involved in the functional differentiation of some cytotoxic T cells (42). As shown in Figure 5G, CD8^+^CD103^+^ cells were higher in db/db mice at the level of all examined tissue (*p* < 0.01).

### Diabetic Mice Show a Redistribution of Naive and Central Memory T Cells From Peripheral Blood (PB) to the BM

**Figure 6:**
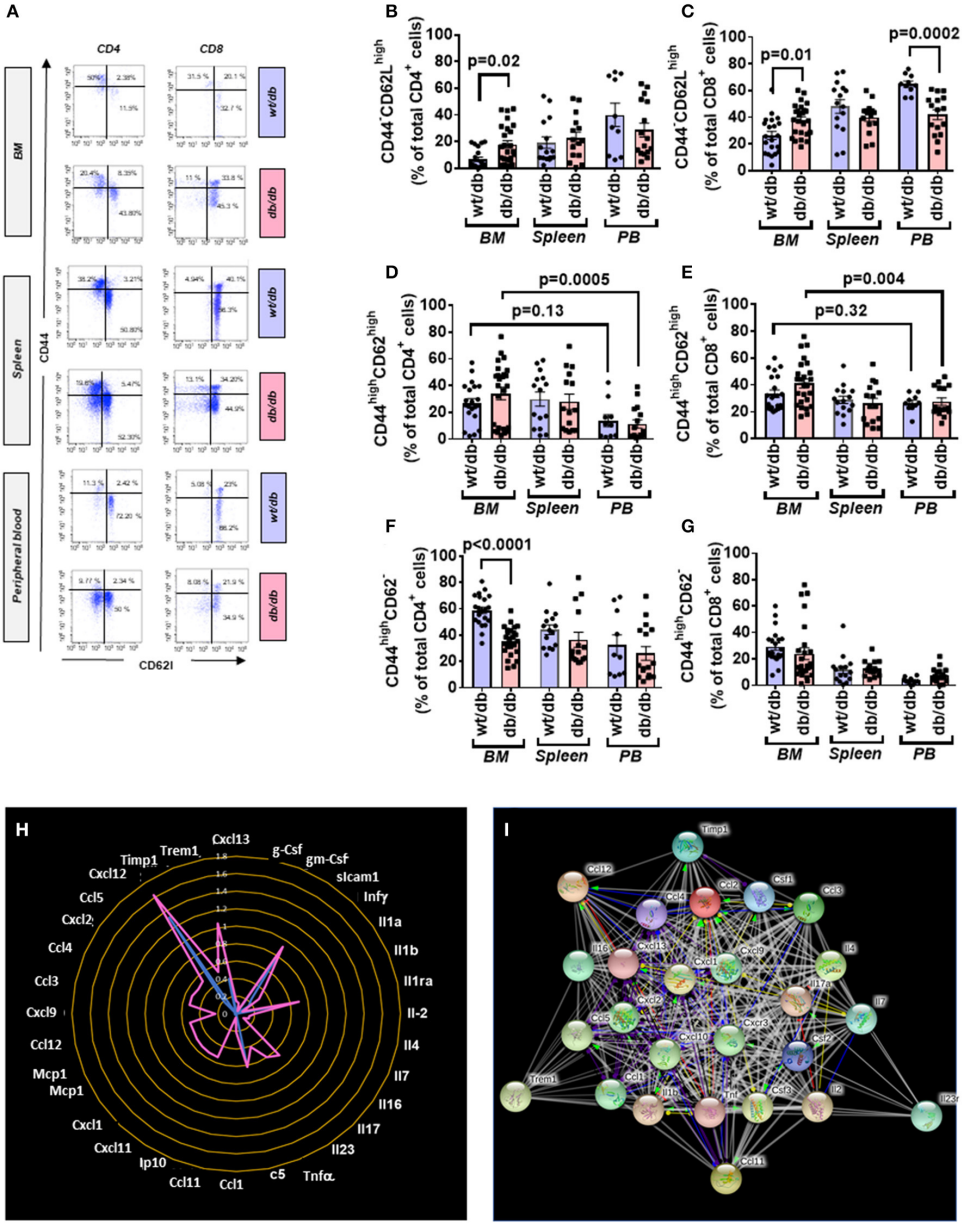
Increased central to peripheral memory cell gradient in diabetic mice. **(A)** Gating strategy for identification of CD44 and CD62L within CD4^+^ and CD8^+^ cells in the BM, spleen, and peripheral blood of wt/db and db/db mice. Reported frequencies are illustrative of a representative case for each group. **(B)** Relative frequency of CD44^−^CD62L^high^ population within CD4^+^ cells. **(C)** Relative frequency of CD44^−^CD62L^high^ population within CD8^+^ cells. **(D)** Relative frequency of CD44^high^CD62L^high^ population within CD4^+^ cells. **(E)** Relative frequency of CD44^high^CD62L^high^ population within CD8^+^ cells. **(F)** Relative frequency of CD44^high^CD62L^−^ population within CD4^+^ cells. **(G)** Relative frequency of CD44^high^CD62L^−^ population within CD8^+^ cells. Values are mean ± SEM, with each point representing an individual case. **(H)** Radar graph of modulated cytokine in BM of db/db mice. Measurements performed in a pool of four mice per group. **(I)** STRING network of modulated factors.

CD4^+^ and CD8^+^ cells were further categorized into naïve and memory phenotypes based on the expression CD44 and CD62L, with the CD44^−^CD62L^high^ population being considered naïve, CD44^high^CD62^high^ population considered TCM, and the CD44^high^CD62L^−^ population considered TEM (Figure 6A).

Naïve cells were increased in BM of db/db mice (CD4^+^: 2.70-fold, *p* = 0.02; CD8^+^: 1.44-fold *p* = 0.01), unaltered in the spleen, and reduced in the peripheral blood (CD4^+^: 0.71-fold, *p* = 0.26; CD8^+^: 0.64-fold, *p* = 0.0002), which resulted in the abrogation of the peripheral to BM gradient seen in the control mice (Figures 6B,C).

Type 2 diabetes on central memory cells did not show differences between groups. Nonetheless, db/db mice manifested a positive gradient between the BM and peripheral blood (CD4^+^: 2.98-fold, *p* = 0.0005; CD8^+^: 1.49-fold, *p* = 0.004) compared with control mice, which did not show a difference between the two districts (CD4^+^: *p* = 0.13; CD8^+^: *p* = 0.32) (Figures 6D,E). CD4^+^ TEM cells were less abundant in BM of db/db mice (0.61-fold, *p* < 0.0001) but unchanged in the other districts (Figure 6F). There was no difference between groups concerning CD8^+^ TEM cells (Figure 6G).

### Altered Cytokine Profile in BM of db/db Mice

Having shown the activation and redistribution of different classes of immune cells in db/db mice, we next examined if this was associated with an alteration of the cytokines content in the BM. Of 40 factors tested, 32 were upregulated in the BM of diabetic mice compared with the wt/db controls. Of these, 22 were uniquely expressed in db/db mice (Figures 6H,I, Table 3). Results indicate the presence of an inflammatory environment in BM, which may account for the recruitment and activation of immune cells.

**Table 3:** Cytokine and chemokines differentially modulated in BM of db/db mice.

**Factor**	**Characteristics**
Ccl2	C-C motif chemokine 2; Chemotactic factor that attracts monocytes, but not neutrophils; Belongs to the intercrine beta (chemokine CC) family!!break (148 aa)
Ccl12	C-C motif chemokine 12; Chemotactic factor that attracts eosinophils, monocytes, and lymphocytes but not neutrophils. Potent monocyte active chemokine that signals through CCR2. Involved in allergic inflammation and the host response to pathogens and may play a pivotal role during early stages of allergic lung inflammation; Belongs to the intercrine beta (chemokine CC) family (104 aa)
Cccl11	Eotaxin; In response to the presence of allergens, this protein directly promotes the accumulation of eosinophils (a prominent feature of allergic inflammatory reactions), but not lymphocytes, macrophages or neutrophils; Belongs to the intercrine beta (chemokine Cnterleukin-4; Participates in at least several B-cell activation processes as well as of other cell types. It is a costimulator of DNA-synthesis. It induces the expression of class II MHC molecules on resting B-cells. It enhances both secretion and cell surface expression of IgE and IgG1. It also regulates the expression of the low affinity Fc receptor for IgE (CD23) on both lymphocytes and monocytes. Positively regulates IL31R
Il4	Interleukin-4; Participates in at least several B-cell activation processes as well as of other cell types. It is a costimulator of DNA-synthesis. It induces the expression of class II MHC molecules on resting B-cells. It enhances both secretion and cell surface expression of IgE and IgG1. It also regulates the expression of the low affinity Fc receptor for IgE (CD23) on both lymphocytes and monocytes. Positively regulates IL31R
Ccl3	3C-C motif chemokine 3; Monokine with inflammatory, pyrogenic and chemokinetic properties. Has a potent chemotactic activity for eosinophils. Binding to a high-affinity receptor activates calcium release in neutrophils; Belongs to the intercrine beta (chemokine CC) fa
Il16	Pro-interleukin-16; Interleukin-16 stimulates a migratory response in CD4+ lymphocytes, monocytes, and eosinophils. Primes CD4+ T-cells for IL-2 and IL-15 responsiveness. Also induces T-lymphocyte expression of interleukin 2 receptor. Ligand for CD4 (1322 aa)
Timp1	Metalloproteinase inhibitor 1; Metalloproteinase inhibitor that functions by forming one to one complexes with target metalloproteinases, such as collagenases, and irreversibly inactivates them by binding to their catalytic zinc cofactor. Acts on MMP1, MMP2, MMP3, MMP7, MMP8, MMP9, MMP10, MMP11, MMP12, MMP13, and MMP16. Does not act on MMP14 (By similarity). Also functions as a growth factor that regulates cell differentiation, migration and cell death and activates cellular signaling cascades via CD63 and ITGB1. Plays a role in integrin signaling; Belongs to the protease
Csf1	Macrophage colony-stimulating factor 1; Cytokine that plays an essential role in the regulation of survival, proliferation and differentiation of hematopoietic precursor cells, especially mononuclear phagocytes, such as macrophages and monocytes. Promotes the release of proinflammatory chemokines, and thereby plays an important role in innate immunity and in inflammatory processes. Plays an important role in the regulation of osteoclast proliferation and differentiation, the regulation of bone resorption, and is required for normal bone development. Required for normal male
Csf2	Granulocyte-macrophage colony-stimulating factor; Cytokine that stimulates the growth and differentiation of hematopoietic precursor cells from various lineages, including granulocytes, macrophages, eosinophils and erythrocytes (141 aa)
Ccl4	C-C motif chemokine 4; Monokine with inflammatory and chemokinetic properties (92 aa)
Cxcl13	C-X-C motif chemokine 13; Strongly chemotactic for B-lymphocytes, weakly for spleen monocytes and macrophages but no chemotactic activity for granulocytes. Binds to BLR1/CXCR5. May play a role in directing the migration of B-lymphocytes to follicles in secondary
Tnf	Tumor necrosis factor; Cytokine that binds to TNFRSF1A/TNFR1 and TNFRSF1B/TNFBR. It is mainly secreted by macrophages and can induce cell death of certain tumor cell lines. It is potent pyrogen causing fever by direct action or by stimulation of interleukin-1 secretion and is implicated in the induction of cachexia, Under certain conditions it can stimulate cell proliferation and induce cell differentiation (235 aa)
Il17a	Interleukin-17A; Ligand for IL17RA. The heterodimer formed by IL17A and IL17F is a ligand for the heterodimeric complex formed by IL17RA and IL17RC (By similarity). Involved in inducing stromal cells to produce proinflammatory and hematopoietic cytokines (B)
Il1b	Interleukin-1 beta; Potent proinflammatory cytokine. Initially discovered as the major endogenous pyrogen, induces prostaglandin synthesis, neutrophil influx and activation, T-cell activation and cytokine production, B-cell activation and antibody production, and fibroblast proliferation and collagen production; Belongs to the IL-1 family (269 aa)
Il2	Interleukin-2; Produced by T-cells in response to antigenic or mitogenic stimulation, this protein is required for T-cell proliferation and other activities crucial to regulation of the immune response. Can stimulate B-cells, monocytes, lymphokine- activated killer cells, n
Cxcl1	Growth-regulated alpha protein; Has chemotactic activity for neutrophils. Contributes to neutrophil activation during inflammation (By similarity). Hematoregulatory chemokine, which, *in vitro*, suppresses hematopoietic progenitor cell proliferation. KC(5-72) shows a highly enhanced hematopoietic activity; Belongs to the intercrine alpha (chemokine CxC) family (96 aa)
Gmcsf3	Granulocyte colony-stimulating factor; Granulocyte/macrophage colony-stimulating factors are cytokines that act in hematopoiesis by controlling the production, differentiation, and function of 2 related white cell populations of the blood, the granulocytes and the monocytes-macrophages. This CSF induces granulocytes; Belongs to the IL-6 superfamily (208 aa)
Trem	Triggering receptor expressed on myeloid cells 1; Stimulates neutrophil and monocyte-mediated inflammatory responses. Triggers release of pro-inflammatory chemokines and cytokines, as well as increased surface expression of cell activation markers. Amplifier of inflammatory responses that are triggered by bacterial and fungal infections and is a crucial mediator of septic shock (By similarity) (230 aa)
Ccl5	C-C motif chemokine 5; Chemoattractant for blood monocytes, memory T-helper cells and eosinophils. Causes the release of histamine from basophils and activates eosinophils. May activate several chemokine receptors including CCR1, CCR3, CCR4, and CCR5. May also be an agonist of the G protein-coupled receptor GPR75. Together with GPR75, may play a role in neuron survival through activation of a downstream signaling pathway involving the PI3, Akt and MAP kinases. By activating GPR75 may also play a role in insulin secretion by islet cells (91 aa)
Cxxl10	C-X-C motif chemokine 10; In addition to its role as a proinflammatory cytokine, may participate in T-cell effector function and perhaps T-cell development; Belongs to the intercrine alpha (chemokine CxC) family (98 aa)
Cxcr3	C-X-C chemokine receptor type 3; Receptor for the C-X-C chemokine CXCL9, CXCL10, and CXCL11 and mediates the proliferation, survival and angiogenic activity of mesangial cells through a heterotrimeric G-protein signaling pathway. Probably promotes cell chemotaxis
Cxcl2	C-X-C motif chemokine 2; Chemotactic for human polymorphonuclear leukocytes but does not induce che
Ccl1	C-C motif chemokine 1; Cytokine that is chemotactic for neutrophils; Belongs to the intercrine beta (chem)
Cxcl9	C-X-C motif chemokine 9; May be a cytokine that affects the growth, movement, or activation state of cells that participate in immune and inflammatory response (126 aa)
Il23r	Interleukin-23 receptor; Associates with IL12RB1 to form the interleukin-23 receptor. Binds IL23 and mediates T-cells, NK cells and possibly certain macrophage/myeloid cells stimulation probably through activation of the Jak-Stat signaling cascade. IL23 functions in innate and adaptive immunity and may participate in acute response to infection in peripheral tissues. IL23 may be responsible for autoimmune inflammatory diseases and be important for tumorigenesis (By similarity) (659 aa)
Il7	Interleukin-7; Hematopoietic growth factor capable of stimulating the proliferation of lymphoid progenitors. It is important for proliferation during certain stages of B-cell maturation (154 aa)

### Rescue of Immune Profile of db/db Mice by Abatacept

Altogether the above findings show a state of inflammation in patients and mice with T2D. This involves activation of adaptive immunity, as evidenced by the increase in CD25^+^ T cells and corroborated by the upregulation of a multitude of inflammation-associated mediators.

**Figure 7:**
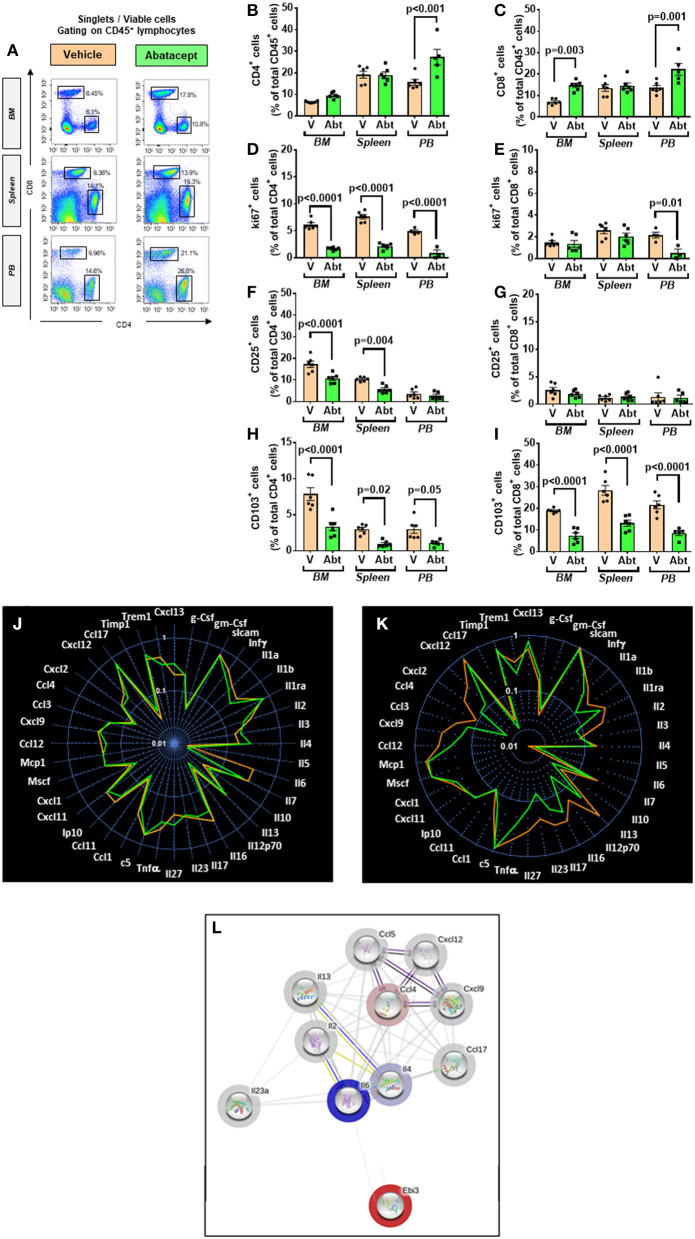
Effect of abatacept on immune cells of T2D mice. **(A)** Gating strategy for identification of CD4^+^ and CD8^+^ cells in the BM, spleen, and peripheral blood of db/db mice given vehicle or abatacept. Reported frequencies are illustrative of a representative case for each group. **(B)** Relative frequency of CD4^+^ cells. **(C)** Relative frequency of CD8^+^ cells. **(D)** Relative frequency of Ki67 expressing cells within CD4^+^ cells. **(E)** Relative frequency of Ki67 expressing cells within CD8^+^ cells. **(F)** Relative frequency of CD25^+^ cells within CD4^+^ cells. **(G)** Relative frequency of CD25^+^ cells within CD8^+^ cells. **(H)** Relative frequency of CD103^+^ cells within CD4^+^ cells. **(I)** Relative frequency of CD103^+^ cells within CD8^+^ cells. **(J)** Spider graph of cytokines in the BM of the abatacept (green line) and vehicle group (orange line). **(K)** Spider graph of cytokines in the peripheral blood of the abatacept (green line) and vehicle group (orange line). Measurements were performed in a pool of four mice per group. **(L)** Network analysis of regulated cytokines. Values are mean ± SEM, with each point representing an individual case.

We next investigated the effect of the *in vivo* administration of abatacept, a CTLA4-Ig fusion protein, on the immune profile of 10-week-old db/db mice. Abatacept- and vehicle-treated (control) mice were sacrificed 4 weeks later. Flow cytometry analysis demonstrated that, compared with control, the abatacept group had higher levels of CD4^+^ and CD8^+^ cells in the peripheral blood (*p* < 0.01 for both comparisons) and, limited to CD8^+^ cells, in BM (*p* = 0.003), whereas no difference was detected in the spleen (Figures 7A–C). The treatment did not affect the abundance of CD19^+^ B lymphocytes (data not shown). These changes in relative cell subsets abundance could be a side effect of changes in chemokine expression after co-stimulation blockade (43). Yet, most importantly, abatacept reduced the proliferation (judged by marker Ki67) of CD4^+^ T cells in the BM, spleen, and peripheral blood, as well of CD8^+^ T cells in the periphery (Figures 7D,E). Corroborating these findings, the levels of activation marker CD25 expressed by CD4^+^ T cells in the BM and the spleen were suppressed by the anti-inflammatory action of abatacept (*p* < 0.01 for both comparisons, Figure 7F) though this was not seen in CD8^+^ T cells (Figure 7G). Moreover, Abatacept reduced the frequency of CD4^+^ and CD8^+^ cells expressing CD103 in all the areas examined (Figures 7H,I).

Examining the cytokine and chemokine production using a Proteome Profiler Mouse Cytokine Array, we did not find any major differences in the BM between abatacept and control-treated animals (Figure 7J). However, in a manner compatible with the systemic effect of abatacept (36), the drug led to a relative reduction in the peripheral blood levels of key classical (type 1) proinflammatory cytokines Il-1b and Il-12, and chemokines Cxcl9, Ccl2/MCP1, Ccl4, and Ccl5, as well as type 17 proinflammatory cytokine Il-17 and Il-23, indicating a clear suppression of type 1 and type 17 proinflammatory signals, as a result of the inhibition of T-cell activation (Figures 7K,L, Table 4).

**Table 4:** Cytokine and chemokine changes induced by Abatacept.

**Factor**	**Value**	**Functions**
Il-27 (Ebi3)	3.98	Il27—Interleukin-27 subunit alpha; Associates with EBI3 to form the IL-27 interleukin, a heterodimeric cytokine which functions in innate immunity. IL-27 has pro- and anti-inflammatory properties, that can regulate T- helper cell development, suppress T-cell proliferation, stimulate cytotoxic T-cell activity, induce isotype switching in B-cells, and that has diverse effects on innate immune cells. Among its target cells are CD4 T-helper cells which can differentiate in type 1 effector cells (TH1), type 2 effector cells (TH2) and IL17 producing helper T-cells (TH17)
Ccl4	3.45	C-C motif chemokine 4; Monokine with inflammatory and chemokinetic properties
Il-13	2.75	Interleukin-13; Cytokine. Inhibits inflammatory cytokine production. Synergizes with IL2 in regulating interferon-gamma synthesis. May be critical in regulating inflammatory and immune responses (By similarity). Positively regulates IL31RA expression in macrophages
Il-23	2.65	Interleukin-23 subunit alpha; Associates with IL12B to form the IL-23 interleukin, a heterodimeric cytokine which functions in innate and adaptive immunity. IL-23 may constitute with IL-17 an acute response to infection in peripheral tissues. IL-23 binds to a heterodimeric receptor complex composed of IL12RB1 and IL23R, activates the Jak- Stat signaling cascade, stimulates memory rather than naive T- cells and promotes production of proinflammatory cytokines. IL-23 induces autoimmune inflammation and thus may be responsible for autoimmune inflammatory diseases
Il-2	2.30	Interleukin-2; Produced by T-cells in response to antigenic or mitogenic stimulation, this protein is required for T-cell proliferation and other activities crucial to regulation of the immune response. Can stimulate B-cells, monocytes, lymphokine- activated killer cells, natural killer cells
Ccl5	2.21	C-C motif chemokine 5; Chemoattractant for blood monocytes, memory T-helper cells and eosinophils. Causes the release of histamine from basophils and activates eosinophils. May activate several chemokine receptors including CCR1, CCR3, CCR4, and CCR5. May also be an agonist of the G protein-coupled receptor GPR75. Together with GPR75, may play a role in neuron survival through activation of a downstream signaling pathway involving the PI3, Akt and MAP kinases
Ccl17	2.13	Chemokine (C-C motif) ligand 17
CXCL12	2.11	Stromal cell-derived factor 1; Chemoattractant active on T-lymphocytes, monocytes, but not neutrophils. Activates the C-X-C chemokine receptor CXCR4 to induce a rapid and transient rise in the level of intracellular calcium ions and chemotaxis. Also binds to atypical chemokine receptor ACKR3, which activates the beta-arrestin pathway and acts as a scavenger receptor for SDF-1. Acts as a positive regulator of monocyte migration and a negative regulator of monocyte adhesion via the LYN kinase. Stimulates migration of monocytes and T- lymphocytes through its receptors, CXCR4
Cxcl9	2.07	C-X-C motif chemokine 9; May be a cytokine that affects the growth, movement, or activation state of cells that participate in immune and inflammatory response
Il-4	2.0	Interleukin-4; Participates in at least several B-cell activation processes as well as of other cell types. It induces the expression of class II MHC molecules on resting B-cells. It enhances both secretion and cell surface expression of IgE and IgG1. It also regulates the expression of the low affinity Fc receptor for IgE (CD23) on both lymphocytes and monocytes. Positively regulates IL31RA expression in macrophages
Il-6	0.02	Interleukin-6; Cytokine with a wide variety of biological functions. It is a potent inducer of the acute phase response. Plays an essential role in the final differentiation of B-cells into Ig- secreting cells Involved in lymphocyte and monocyte differentiation. Acts on B-cells, T-cells, hepatocytes, hematopoietic progenitor cells and cells of the CNS. Required for the generation of T(H)17 cells. Also acts as a myokine. It is discharged into the bloodstream after muscle contraction and acts to increase the breakdown of fats and to improve insulin resistance

**Figure 8:**
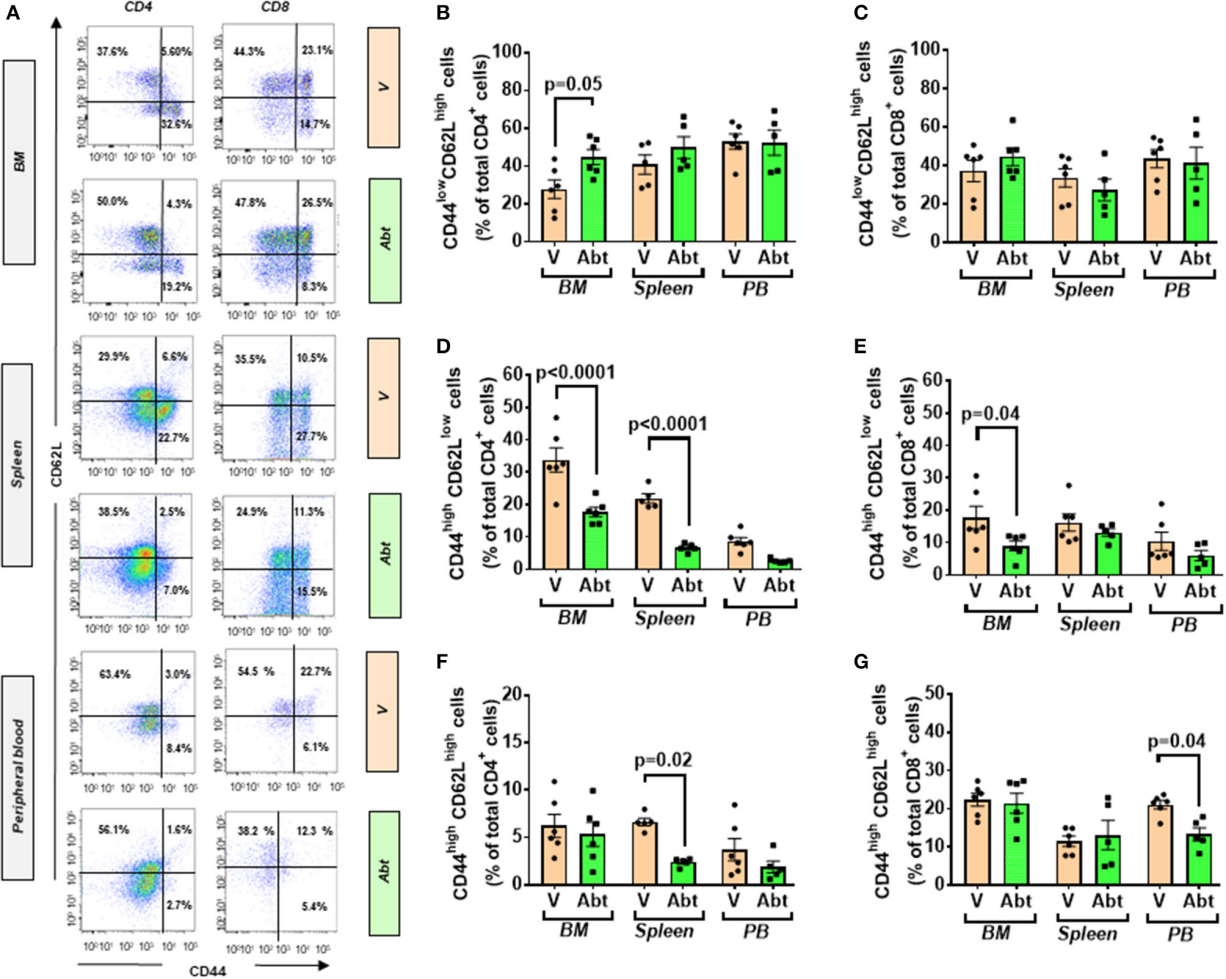
Effect of abatacept on naïve and memory T cells of T2D mice. **(A)** Gating strategy for identification of CD44^+^ and CD62L^+^ within CD4^+^ and CD8^+^ fractions in the BM, spleen, and peripheral blood of db/db mice given vehicle or abatacept. Reported frequencies are illustrative of a representative case for each group. **(B)** Relative frequency of naïve cells within CD4^+^ cells. **(C)** Relative frequency of naïve cells within CD8^+^ cells. **(D)** Relative frequency of TEM cells within CD4^+^ cells. **(E)** Relative frequency of TEM cells within CD8^+^ cells. **(F)** Relative frequency of TCM cells within CD4^+^ cells. **(G)** Relative frequency of TCM cells within CD8^+^ cells. Values are mean ± SEM, with each point representing an individual case.

We further analyzed the abundance of APCs. Abatacept decreased the number of DC in peripheral blood (3.3 ± 0.4 vs. 7.3 ± 1.2% in vehicle, *p* < 0.05) but did not alter the frequency of macrophages (data not shown). Finally, we examined the effect of abatacept on naïve and memory T cells (Figure 8A). Regarding naïve cells, the abatacept-treated group showed a borderline increase of the CD4^+^ fraction in BM (*p* = 0.05), whereas naïve CD8^+^ cells were unaltered (Figures 8B,C). CD4^+^ TEM cells were reduced by abatacept in BM and spleen (*p* < 0.0001 for both comparisons), whereas CD8^+^ TEM cells were decreased only in BM (*p* = 0.04). Figures 8D,E). TCM cells followed a more heterogeneous behavior, the CD4^+^ fraction being reduced in the spleen (*p* = 0.02) and the CD8^+^ fraction in the peripheral blood (*p* = 0.04) (Figures 8F,G).

### Metabolic and Functional Endpoints

**Figure 9:**
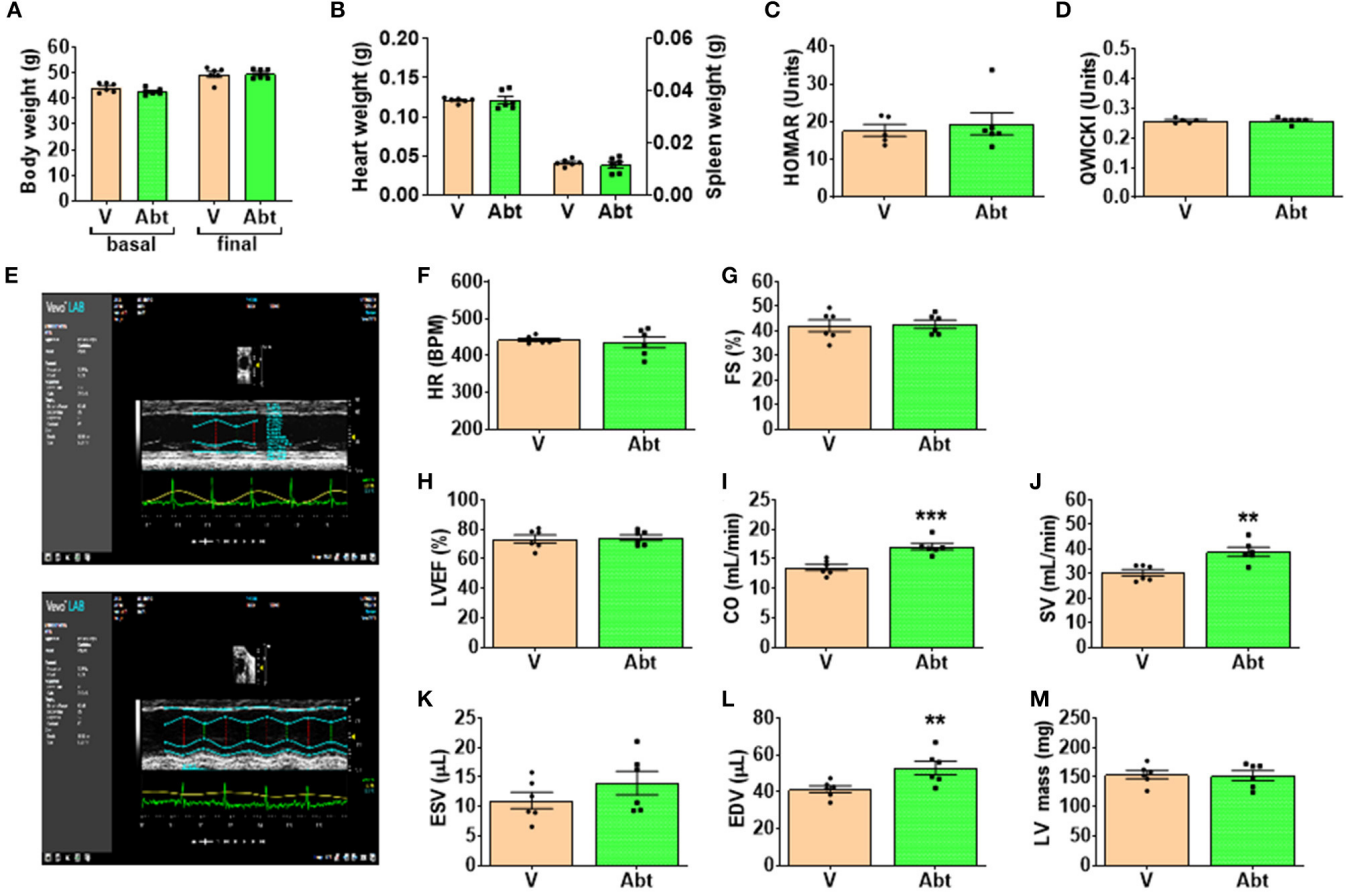
Effect of abatacept on metabolic and cardiac endpoints. **(A)** Body weight, **(B)** Heart weight, **(C,D)** indices of insulin sensitivity, and **(E–M)** indices of cardiac function. Values are mean ± SEM, with each point representing an individual case. **p < 0.01, and ***p < 0.001 vs. vehicle (V).

Body weight and the weight of heart and spleen were similar between groups given vehicle or abatacept (Figures 9A,B). Likewise, there was no difference in the fasting levels of insulin and glucose (data not shown), as well as in indexes of insulin sensitivity HOMA-IR and QUICKI (Figures 9C,D). We then analyzed the cardiac phenotype by echocardiography. The study was conducted at an age when db/db mice manifest echocardiographic evidence of left ventricular (LV) dysfunction (44). abatacept induced an improvement in echocardiographic indexes of systolic function compared with controls (Figures 9E–G). Specifically, compared with the control group, abatacept-treated mice showed higher cardiac output (CO, *p* < 0.001) and stroke volume (SV, *p* < 0.01), which could be reconducted to an increased preload, as suggested by the increased LV end diastolic volume (EDV, *p* < 0.01) (Figures 9H–M). However, abatacept did not reduce the LV mass, thus discarding an anti-hypertrophic effect of the drug.

## Discussion

Recent evidence indicates that T cells play key roles in the progression of T2D. *In vitro* studies on immune cells harvested from the peripheral blood of subjects with T2D and ND controls have shown that T2D is associated with overactivated T cells and the induction of the inflammatory pathways (45–47), thus positing the provoking hypothesis of T2D being an autoimmune disease (48). The role of BM in the maintenance of antigen-experienced adaptive immune cells is well-documented as reviewed in Bonomo et al. (49). Nonetheless, little is known about the immune profile of BM in T2D.

In the present study, we provide novel proof-of-concept evidence for the activation of adaptive immunity in the BM of patients and mice with T2D. The studied human diabetic cohort had an increased frequency of BM T lymphocytes. Both CD4^+^ and CD8^+^ cells abundantly expressed CCR7, which, together with the observed high levels of CCL19 (CCR7 receptor ligand), suggests that the local BM environment was reshaped to favor immune cell recruitment from the circulation. Moreover, we demonstrated that the diabetic BM contains TCM cells that have undergone recent activation. Most importantly, BM T cells showed a clear upregulation of typical activation markers. Several features of the adaptive immunity activation seen in patients were confirmed in db/db mice. Finally, we proved that the treatment with abatacept, which interferes with the co-stimulation signaling mechanism, inhibited the activation of adaptive immunity, and improved cardiac function in db/db mice.

Abundant fat depots of overweight/obese patients reportedly contain autoreactive T cells that exert a cytotoxic effect on adipocytes and fuel adipose inflammation (50, 51). Adipose tissue is also contiguous with main lymphoid organs, such as lymph nodes, thymus, and BM, participating in multiple intertwined mechanisms (52). For instance, adipocytes surrounding the thymus may influence T-cell differentiation in response to metabolic challenges (53). Likewise, adipocytes that reside in the BM are thought to play relevant roles in hematopoiesis, lymphopoiesis, and memory B- and T-cell responses (21). BM adiposity becomes more abundant with aging, in parallel with a decline of immunity and hematopoietic functions (54). Fat accumulation reported by us in the BM of patients and animal models with T2D represents a significant difference (25, 27, 55). In fact, the analysis of BM cells indicates a reactive adaptive immunity. These new data complement our previous report showing cytotoxic NK cells were significantly increased in the BM of subjects with T2D (25). The cellular phenotype reported in the present study was associated with alterations in the levels of cytokines and chemokines that influence the lodging and activation of naïve T cells and the maintenance of a memory reservoir. It remains to be ascertained whether adaptive immunity was induced by the previous exposure to foreign antigens or could be reconducted to dysregulation of co-signaling mechanisms that direct T-cell function and fate following antigen presentation. Interestingly, we found that CD147 was upregulated in the diabetic BM. This immunoglobulin acts as the main upstream stimulator of matrix metalloproteinases and may have pathogenic roles in diabetic complications, through the recruitment of immune cells. Intriguingly, CD147 is considered a key route for severe acute respiratory syndrome coronavirus 2 (SARS-CoV-2) invasion of immune cells (38), which might account for the susceptibility of diabetic patients to viral infection (56).

The co-signaling factor CTLA4 exerts competitive inhibition of T-cell activation (40). We tested the importance of this signaling mechanism by treating T2D mice with abatacept, a CTLA4-Ig fusion protein that exerts anti-inflammatory activity and attenuates T-cell activation by inhibiting the CD80/86:CD28 co-stimulatory pathway. The murine CD28 has a 77% homology with human CD28 (https://www.genecards.org/cgi-bin/carddisp.pl?gene=CD28). Moreover, this immunomodulatory drug showed cardioprotective effects in mice with pressure overload-induced heart failure (36). In our study abatacept inhibited T-cell activation and suppressed the production of several key inflammatory cytokines and chemokines. Treg cells are sensitive to co-stimulation perturbations, and it cannot be excluded that the reduction in CD25 expressing T cells following abatacept treatment is due to the effects on Treg. This possibility warrants focused future investigation.

The clinical indication of abatacept is to treat active rheumatoid arthritis, but seminal data are supporting its potential in the treatment of Type 1 diabetes and cardiovascular disease. In TrialNet study for people newly diagnosed, those who took Abatacept showed 59% higher insulin production and prolonged insulin production (57). Moreover, clinical studies in patients with rheumatoid arthritis have shown that Abatacept treatment reduced the risk of Type 1 or Type 2 diabetes, through an anti-inflammatory mechanism preserving β-cell function (33–35). A recent case report showed abatacept treatment led to the resolution of glucocorticoid-refractory myocarditis caused by rheumatoid arthritis or immune checkpoint inhibitor anti-cancer treatment (58). Here, we showed that, although not capable of improving insulin resistance, the anti-inflammatory action of abatacept could benefit the cardiac function of db/db mice.

## Conclusion

In the present study, we have shown the activation of adaptive immunity in diabetic BM and the possibility to rescue immune and cardiac abnormalities using the clinically available, immunomodulatory drug abatacept. The compound is not risk free and the use in diabetes requires additional investigation assessing safety and optimal risk-to-benefit context. Regarding limitations, we could not perform analyses of peripheral blood cells in the human study because the ethical license covered the investigation of BM leftovers only. The focus of the present study was on specific populations of lymphocytes, which were found generally increased/activated. This change implies a relative reduction in other BM cell populations expressing the CD45 marker, an aspect that was not assessed in the present study but documented previously by us and others (25, 30, 59, 60). Considering the pilot nature of the work, further investigation is necessary in large cohorts of patients to determine whether the activation of adaptive immune cells in BM is a general phenomenon or characterizes specific subgroups of diabetic patients according to disease duration and severity of complications. Moreover, an additional mechanistic investigation is warranted on the causes responsible for the concomitant T-cell activation and expansion of naïve T cells in BM, the latter likely due to the increased homing from secondary lymphoid organs. Finally, studies in models of diet-induced diabetes could strengthen the novel evidence provided by data from the genetically diabetic db/db mice.

## Data Availability Statement

The raw data supporting the conclusions of this article will be made available by the authors, without undue reservation.

## Ethics Statement

The studies involving human participants were reviewed and approved by Bristol NHS. The patients/participants provided their written informed consent to participate in this study. The animal study was reviewed and approved by UK Home Office.

## Author Contributions

MS performed the flow cytometry studies and wrote the draft of the paper. NS and AB were involved in patient recruitment and sample collections. AT was responsible for the *in vivo* studies. VA performed the metabolic assays. LN and MK provided expert opinion on protocols and interpretation of immune responses. GS contributed to the analysis and interpretation of data. PM elaborated on the scientific hypothesis, wrote the final version, procured financial support, was the guarantor of this work, had full access to all of the data in the study, and took responsibility for the integrity of the data and the accuracy of the data analysis. All authors contributed to the article and approved the submitted version.

## Conflict of Interest

The authors declare that the research was conducted in the absence of any commercial or financial relationships that could be construed as a potential conflict of interest.

## Abbreviations

APCs: antigen-presenting cells
BM: bone marrow
BMMCs: BM mononuclear cells
CO: cardiac output
TCM: central memory T cells
CCR7: CC-chemokine receptor 7
DCs: dendritic cells
EDV: end diastolic volume
TEM: effector memory T cells
ND: non-diabetic
HSPCs: hematopoietic stem/progenitor cells
SV: stroke volume
TEMRA: terminally differentiated T memory cells
T2D: type 2 diabetes
VAT: visceral adipose tissue
PBS: phosphate buffered saline
RT: room temperature
EDTA: ethylenediaminetetraacetic acid
RT: room temperature
FACS: flow cytometry staining
RPMI: Roswell Park Memorial Institute
RBS: red blood cell
SARS-CoV-2: severe acute respiratory syndrome coronavirus 2.
